# Nonlinear model identification and spectral submanifolds for multi-degree-of-freedom mechanical vibrations

**DOI:** 10.1098/rspa.2016.0759

**Published:** 2017-06-14

**Authors:** Robert Szalai, David Ehrhardt, George Haller

**Affiliations:** 1 Department of Engineering Mathematics, University of Bristol, Merchant Venturers Building, Woodland Road, Bristol BS8 1UB, UK; 2 Department of Mechanical Engineering, University of Bristol, Queen’s Building, University Walk, Clifton BS8 1TR, UK; 3 Institute for Mechanical Systems, ETH Zürich, Leonhardstrasse 21, Zürich 8092, Switzerland

**Keywords:** nonlinear normal modes, model identification, nonlinear vibrations, invariant manifolds

## Abstract

In a nonlinear oscillatory system, spectral submanifolds (SSMs) are the smoothest invariant manifolds tangent to linear modal subspaces of an equilibrium. Amplitude–frequency plots of the dynamics on SSMs provide the classic backbone curves sought in experimental nonlinear model identification. We develop here, a methodology to compute analytically both the shape of SSMs and their corresponding backbone curves from a data-assimilating model fitted to experimental vibration signals. This model identification utilizes Taken’s delay-embedding theorem, as well as a least square fit to the Taylor expansion of the sampling map associated with that embedding. The SSMs are then constructed for the sampling map using the parametrization method for invariant manifolds, which assumes that the manifold is an embedding of, rather than a graph over, a spectral subspace. Using examples of both synthetic and real experimental data, we demonstrate that this approach reproduces backbone curves with high accuracy.

## 1. Introduction

Modal decomposition into normal modes is a powerful tool in linear system identification [1], but remains inapplicable to nonlinear systems due to the lack of a superposition principle. Various nonlinear normal mode (NNM) concepts nevertheless offer a conceptual simplification in the description of small-amplitude nonlinear vibrations.

For conservative oscillatory systems with no resonance, the Lyapunov subcentre-manifold theorem [2] guarantees the existence of a unique, analytic surface of periodic orbits that is tangent to any selected two-dimensional modal subspace (or eigenspace) of the linearized system at the equilibrium. Each periodic orbit in such a subcentre manifold is an NNM by the classic definition of Rosenberg [3]. By contrast, Shaw & Pierre [4] call the subcentre manifold itself an NNM.

Shaw & Pierre [4] also extend the latter view to dissipative systems, envisioning NNMs as invariant manifolds tangent to modal subspaces of an equilibrium point (see the reviews in [5–9]). As observed recently, however, by multiple authors [10–12], such invariant manifolds are non-unique even for linear systems, let alone for nonlinear ones. Formal Taylor expansions and operational numerical procedures do nevertheless yield approximate invariant surfaces in most problems. This effectiveness of the Shaw–Pierre approach has inspired its formal extension to invariant manifolds modelled over multiple modes [13], as well as to time-dependent invariant manifolds under external harmonic forcing [14,15].

**Figure 1.**
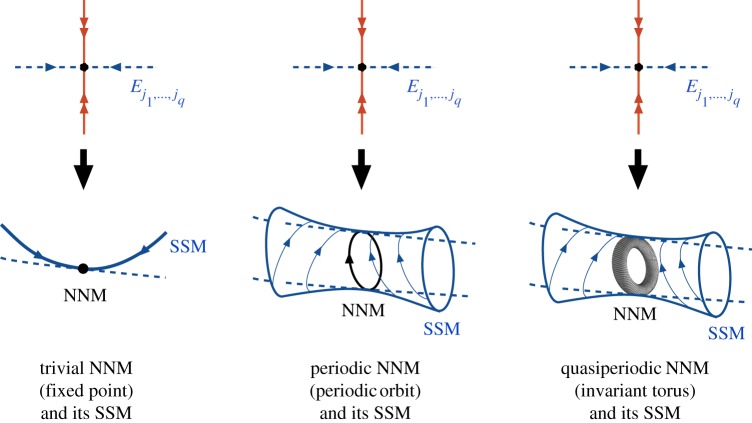
The three main types of NNMs (trivial, periodic and quasi-periodic) and their corresponding SSMs (autonomous, periodic and quasi-periodic). The NNMs are, or are born out of perturbations of, a fixed point. The SSMs are, by contrast, the smoothest invariant manifolds tangent to a subbundle along the NNM whose fibres are close to a specific modal subspace *E*_*j*_1_,…,*j*_*q*__ of the linearized system. Here, the indices *j*_1_,…,*j*_*q*_ refer to an arbitrary selection of *q* two-dimensional modal subspaces of the linearized system (cf. [12] for more details). (Online version in colour.)

In a recent mathematical treatment, Haller & Ponsioen [12] unites the Rosenberg and Shaw–Pierre NNM concepts for dissipative systems under possible time-dependent forcing. In this setting, a NNM is a near-equilibrium oscillation with finitely many frequencies. This NNM concept includes the trivial case of an equilibrium with no (i.e. zero) oscillation frequencies; Rosenberg’s case of a periodic orbit; and the case of a quasi-periodic oscillation with finitely many rationally independent frequencies. Haller & Ponsioen [12] then define a SSM as the smoothest invariant manifold tangent to a spectral subbundle along an NNM. For a trivial NNM (equilibrium), a spectral subbundle is a modal subspace of the linearized system at the equilibrium, and hence an SSM is the smoothest Shaw–Pierre-type invariant manifold tangent to this modal subspace. Similarly, for periodic or quasi-periodic NNMs, an SSM is the smoothest invariant manifold among those sought formally in time-dependent extensions of the Shaw–Pierre surfaces (figure 1).

Here, we adopt the above distinction between NNMs and SSMs and restrict our attention to SSMs of trivial NNMs (i.e. zero-amplitude periodic orbits). Even in this simplest setting, it is not immediate that a single smoothest invariant manifold tangent to a modal subspace of the fixed point actually exists. This question, however, is positively answered under certain conditions by the abstract invariant manifold results of Cabré *et al.* [16], as explained by Haller & Ponsioen [12]. These results also provide a computationally efficient way of computing SSMs using the parametrization method (cf. [17] for a general introduction).

The reduced dynamics on a single-mode SSM gives an exact nonlinear extension of the linear dynamics of the modal subspace to which the SSM is tangent. This extension is characterized by a *backbone curve,* i.e. a graph expressing the instantaneous vibration amplitude as a function of the instantaneous vibration frequency along the SSM.

Without specific concern for SSMs, backbone curves have been approximated operationally in a number of numerical studies. One approach assumes that the mechanical system is conservative apart from a weak damping term that is a linear (or at least odd), position-independent function of the velocities. In such a system, a periodic forcing producing a 90^°^ out-of-phase response preserves exactly a periodic orbit (i.e. Rosenberg’s NNM) of the conservative limit [18,19]. The systematic construction of external forcing that yields the required 90^°^ phase lag for various frequencies is usually referred to as the *force appropriation method*. In practice, force appropriation involves a tedious tuning process that also suffers from unintended interactions between a shaker and the nonlinear system.

To expedite the backbone curve construction, one may locate a single high-amplitude periodic NNM from force appropriation, then turn off the forcing, and identify, by signal processing, the instantaneous amplitude–frequency relation of the decaying vibration as the backbone curve. Usually referred to as the *resonance decay method*, this process tacitly assumes that the decaying vibrations closely follow a Lyapunov subcentre manifold of the conservative limit. In our terminology, the assumption is that the analytic subcentre manifold of the conservative limit perturbs smoothly to a unique SSM under small enough damping. While this statement seems exceedingly difficult to establish mathematically, it appears to hold true for small enough viscous damping [20,18]. Therefore, under weak viscous damping, the resonance decay approach gives consistent results for SSMs, provided that the decaying vibrations are close to the (yet unknown) SSM. Small errors in this initialization are expected to be persistent for fast SSMs, i.e. SSMs tangent to the modal subspaces with higher damping. This is because the off-SSM components (errors) in the initial conditions decay much slower than the in-SSM components (useful signal), which adds substantial inaccuracy to the backbone curve construction.

A third approach to backbone-curve construction uses time-dependent normal forms to construct approximate reduced-order nonlinear models of the system near each natural frequency. The backbone curve is then obtained approximately by the method of harmonic balance applied to the reduced model under resonant parametric forcing [10]. An advantage of this method is its ability to deal with internal resonances, producing intricate multi-dimensional backbone surfaces. The underlying assumption for all this is that the higher-order normal form terms coupling the reduced model to the remaining degrees of freedom are small, and that the oscillations have small enough amplitudes for the harmonic balance method to be reasonably accurate. A further important assumption is that an exact nonlinear model for the system is available for the purposes of computing a normal form. This tends to limit the practical use of this approach to simple geometries and materials.

In summary, several methods for numerical or experimental backbone-curve construction are available, but all make assumptions limiting their range of applicability. These assumptions include small, position-independent and linear viscous damping; small enough oscillation amplitudes; an accurate initial knowledge of the SSM; and yet unproven results on the smooth persistence of Lyapunov subcentre manifolds as SSMs under non-zero damping.

Here, we develop a backbone-curve identification method that addresses most of the above challenges. We infer backbone curves directly from the dynamics on the SSM, without making any assumption on the type and magnitude of the damping, or relying on any (yet unproven) relation between dissipative NNMs and the Lyapunov subcentre manifold of the conservative limit. We focus on SSMs of equilibria and hence assume no external forcing throughout the paper. As input, we assume that tracks of decaying vibration data are available in the vicinity of *N* natural frequencies. We simultaneously assimilate all these data into a nonlinear discrete mapping model of the near-equilibrium dynamics of the system. We then construct backbone curves analytically from the nonlinear dynamics on the SSMs of this discrete mapping.

The term *resonance* in this paper will always refer to algebraic conditions among eigenvalues of the equilibrium, and hence is unrelated to external forcing. Specifically, an*internal resonance* will refer to integer linear combinations of eigenvalues inside a spectral subspace E tangent to an SSM through a fixed point. Similarly, *an external resonance* will refer to integer linear combinations of eigenvalues inside a spectral subspace E with eigenvalues outside E. External resonance conditions turn out to determine the existence of an SSM, while internal resonances dictate the dynamics within the SSM, and hence govern the form of the backbone curve associated with the SSM.

We illustrate the generality and accuracy of this approach on two examples. Our first example is a two-degree-of-freedom nonlinear mechanical system, for which we perform both an analytic and a data-assimilating construction of the backbone curves for comparison. Our second example is a clamped–clamped beam experiment [21], in which we determine the first three SSMs simultaneously from measurements of decaying vibration signals.

## 2. Set-up

We start with an *n*-degree of freedom, autonomous mechanical system of the general form

2.1 $$\text{M}(\text{q})\ddot{\text{q}}-\text{f}(\text{q},\dot{\text{q}})=\text{0},\;\text{f}(\text{0},\text{0})=\text{0},$$

where the mass matrix $\text{M}(\text{q})\in R^{n\times n}$ and its inverse ***M***^−1^(***q***) are of class *C*^*r*^, with *r*≥1, in the generalized coordinate vector $\text{q}\in R^{n}$. The forcing vector $\text{f}(\text{q})\in R^{n}$ is also *C*^*r*^ in its arguments, containing all conservative and non-conservative autonomous forces, both linear and nonlinear.

Beyond taking non-negative integer values, the smoothness parameter *r* is also allowed to be r=∞ (arbitrarily many times differentiable functions) or *r*=*a* (analytic functions, i.e. $C^{\infty }$ functions with a convergent Taylor expansion in a complex neighbourhood of $\left(\text{q},\dot{\text{q}})=(\text{0},\text{0}\right)$). The degree of freedom *n*≥1 is allowed to be arbitrarily high and may also be in principle infinity (continuum vibrations), although some of our assertions about properties of the solutions would need to be verified on a case-by-case basis in the infinite-dimensional setting. By the formulation in (2.1), ***q***≡**0** is an equilibrium point for the system.

The equivalent first-order form of the differential equation (2.1) is obtained by letting $\text{x}=(\text{q},\dot{\text{q}})\in R^{2n},$ which leads to

2.2 $$\dot{\text{x}}=F(\text{x}),\;F(\text{0})=\text{0},\;F(\text{x})=\left(\begin{array}{c} \dot{\text{q}}\\ {\text{M}}^{-1}(\text{q})\text{f}(\text{q},\dot{\text{q}}) \end{array}\right),$$

where $F(\text{x})\in R^{2n}$ is *C*^*r*^ in its arguments. The solutions ***x***(*t*) of (2.2) give rise to the flow map

$$\Xi_{t}:{\text{x}}_{0}\mapsto \text{x}(t),$$

where ***x***_0_=***x***(0).

The linearization of (2.2) at the equilibrium point ***x***=**0** is given by

2.3 $$\dot{\text{x}}=A\text{x},\;A=DF(\text{0}),$$

where symbol *D* stands for differentiation, We assume that A has *n* pairs of complex conjugate eigenvalues $\lambda_{1},{\bar{\lambda }}_{1},\ldots ,\lambda_{n},{\bar{\lambda }}_{n}$, satisfying

2.4 Reλn≤⋯≤Re λ1<0,

and hence the equilibrium point is linearly asymptotically stable. This context is relevant for underdamped structural vibrations, in which the nonlinear system (2.1) is known to have a stable equilibrium, but the exact nature of its nonlinearities is unknown.

Note that vanishing linearized frequencies (i.e. rigid-body modes for the linearized system in the absence of damping) pose no problem for our forthcoming analysis, as long as these modes are linearly damped, as ensured by our main assumption (2.4).

## 3. Sampled nonlinear vibrations

To reduce the complexity of the flow map ***Ξ***_*t*_ in our analysis, we will focus on temporally sampled approximations to ***Ξ***_*t*_. Iterating such discrete approximations, one can still reproduce the main features of the nonlinear dynamics at regular time intervals. Constructing the sampled dynamics via a stroboscopic (or Poincaré) map is, however, only feasible when the full dynamical system (2.1) is precisely known, and hence trajectories from arbitrary initial conditions can be generated. In practice, this is generally not the case.

Instead, we seek to reconstruct a sampled representation of ***Ξ***_*t*_ from a limited number of observations of trajectories. The scalar observable along trajectories can be, for instance, a position or a velocity coordinate of a certain material point of the mechanical system (2.1). We denote this observable by $\varphi (\text{x}):R^{2n}\to R$, i.e. as a scalar function of the state variable ***x*** alone. We then build a new state vector $\xi \in R^{2\nu }$ out of 2*ν* subsequent observations along trajectories of (2.1) by letting

3.1 $$\xi =\Phi (\text{x}),\;\Phi (\text{x}):=(\varphi (\text{x}),\varphi (\Xi_{T}(\text{x})),\ldots ,\varphi (\Xi_{T}^{2\nu -1}(\text{x})))\in R^{2\nu },\;\nu \geq 1.$$

We have selected the dimension of ***ξ*** to be even (i.e. 2*ν*) to ensure basic spectral compatibility between the dynamics of ***ξ*** and the dynamics of ***x***, as discussed in more detail below.

A sampling map ***F*** can be defined as the discrete mapping advancing the current 2*ν* observations by one, i.e. from the observation vector ***Φ***(***x***) to the observation vector ***Φ***(***Ξ***_T_(***x***)). Specifically, we define the mapping $\text{F}:R^{2\nu }\to R^{2\nu }$ via the relation

3.2 $$\xi_{k+1}:=\text{F}(\xi_{k})={\text{F}}^{k}(\xi_{0}),\;k\in N,\;\xi_{0}=\Phi ({\text{x}}_{0}),$$

or, equivalently, as

3.3 $$\Phi \circ \Xi_{T}=\text{F}\circ \Phi .$$

By construction, the ***x***=0 equilibrium point of system (2.1) is mapped into a fixed point ***ξ*^0^**=*Φ*(0) of the sampling map ***F*** under ***Φ***. If necessary, we shift the ***ξ*** coordinates as ***ξ***→***ξ***−***ξ*^0^** to achieve ***ξ*^0^**=**0**. Therefore, without loss of generality, we may assume

3.4 F(0)=0.

As a consequence, whenever *φ*∈*C*^*r*^ holds, a Taylor expansion of ***F*** at the origin must be of the form

3.5 $$\text{F}(\text{x})=\sum_{|\text{m}|=1}^{r}{\text{a}}_{\text{m}}\xi_{1}^{m_{1}}\cdot \cdots \cdot \xi_{2\nu }^{m_{2\nu }}+o(|\text{x}{|}^{r})=\sum_{|\text{m}|=1}^{r}{\text{a}}_{\text{m}}\xi^{\text{m}}+o(|\text{x}{|}^{r})$$

for appropriate coefficient vectors ${\text{a}}_{\text{m}}\in R^{2\nu }$ and integer index vector $\text{m}=(m_{1},\ldots ,m_{2\nu })\in N^{2\nu }$, whose norm we measure as $|\text{m}|=\sum_{i=1}^{2\nu }m_{i}.$ We have used here the short-hand notation $\xi^{\text{m}}=\xi_{1}^{m_{1}}\cdot \cdots \cdot \xi_{2\nu }^{m_{2\nu }}$.

## 4. Delay embedding

The definition (3.2) does not immediately clarify the relation between the dynamics of the flow map ***Ξ***_T_ and the dynamics of the sampling map ***F***. The Takens Embedding Theorem [22], however, guarantees that such a relationship exists on invariant manifolds of generic flow maps ***Ξ***_T_, at least for generic observables *φ*, as long as the sample length 2*ν* is long enough.

Specifically, if *W* is a compact, *d*-dimensional inflowing-invariant manifold [23] of system (2.1) and 2*ν*≥2*d*+1 holds, then the set of function pairs (***Ξ***_T_,*φ*) for which ***Φ***(*W*) is diffeomorphic to *W* is open and dense in the product space $D^{r}(W)\times C^{r}(W,R).$ Here, $D^{r}(W)$ denotes the space of *C*^*r*^ diffeomorphisms of *W*, and $C^{r}(W,R)$ denotes the space of *C*^*r*^ scalar functions defined on *W*, with both spaces endowed with the *C*^*r*^ topology.

Takens’s theorem can further be strengthened [24,25] when ***Ξ***_T_ has only a finite number of periodic orbits of periods less than 2*ν*, with all periodic orbits admitting distinct Floquet multipliers. In this case, for *any* ***Ξ***_T_, there is an open and dense set of observables $\varphi \in C^{r}(W,R)$ such that ***Φ*** is an embedding of *W* into $R^{2\nu }$. This version of the theorem is particularly helpful in our setting, as close enough to its asymptotically stable equilibrium at ***x***=0, the flow map ***Ξ***_T_ will have no periodic orbits. Therefore, it is enough for us to require the observable *φ* to be generic, without having to assume anything further for ***Ξ***_T_. This simplification holds true on any extended neighbourhood of the origin that has the required low number of nondegenerate periodic orbits discussed above.

For such generic observables, $\Phi (W)\subset R^{2\nu }$ is a diffeomorphic copy of the invariant manifold $W\subset \;R^{2n}$. Importantly, ***Φ***(*W*) is then an invariant manifold for the discrete dynamical system (3.2) by definition. On this invariant manifold, the map ***F*** is conjugate to the flow map ***Ξ***_T_ by formula (3.3), which can now be rewritten as

4.1 $$\text{F}=\Phi \circ \Xi_{T}\circ \Phi^{-1}:\Phi (W)\to \Phi (W)$$

given that ***Φ*** is a diffeomorphism onto its image.

Consequently, any coordinate-independent dynamical feature of ***Ξ***_T_ will be shared by the mapping ***F***. This will be a crucial point in our strategy to build a faithful reduced-order model for system (2.1). Specifically, we will use an experimentally observed scalar *φ* to approximate the Taylor expansion (3.5) of the mapping ***F***.

Our focus here is the reconstruction of the dynamics of ***F*** on two-dimensional invariant manifolds *W* tangent to two-dimensional modal subspaces of the linearized flow map *D****Ξ***_T_(**0**) at the equilibrium point. We thus have *d*=2, and hence the minimal dimension for the embedding space $R^{2\nu }$ required by Takens’s theorem is 2*ν*≥5, implying *ν*≥3 (For our first example of a two-degree-of-freedom model in §9a, a comparison with exact analytic computation shows that a reconstruction with *ν*=2 already suffices, but this cannot be generally guaranteed.)

The tangent space *T*_0_*W* of *W* at the origin is a two-dimensional invariant subspace for *D****Ξ***_T_(0). Specifically, *T*_0_*W* is the modal subspace corresponding to a pair of complex conjugate eigenvalues $\left(\mu_{\ell },{\bar{\mu }}_{\ell })=(e^{\lambda_{\ell }T},e^{{\bar{\lambda }}_{\ell }T}\right)$, where $\left(\lambda_{\ell },{\bar{\lambda }}_{\ell }\right)$ are eigenvalues of A, ordered as in (2.4). The conjugacy relationship (4.1) and formula (3.4) then imply that $\left(\mu_{\ell },{\bar{\mu }}_{\ell }\right)$ are also eigenvalues of the linearized sampling map *D****F***(**0**) at ***ξ***=**0**, i.e. we have

4.2 $$\{\mu_{\ell },{\bar{\mu }}_{\ell }\}=\{e^{\lambda_{\ell }T},e^{{\bar{\lambda }}_{\ell }T}\}\subset \mathrm{Spect}\{D\text{F}(0)\},$$

where *Spect*{*D****F***(**0**)} denotes the spectrum (i.e. set of eigenvalues) of the Jacobian matrix *D****F***(**0**).

## 5. Spectral submanifolds of the sampling map

The linearized sampled dynamics near the fixed point ***ξ***=**0** is governed by the Jacobian ***A***=*D****F***(**0**) of the sampling map ***F***. We assume that this Jacobian is diagonalizable and collect its complex eigenvectors in a matrix $\text{V}\in C^{2\nu \times 2\nu }$. Introducing the new coordinate $\text{y}\in C^{2\nu }$ via the relation

5.1 ξ=Vy,

we obtain the transformed form of (3.2) as

5.2 $$\begin{array}{rl} {\text{y}}_{k+1} & =\Lambda {\text{y}}_{k}+\text{G}({\text{y}}_{k}),\;\Lambda =\mathrm{diag}(\mu_{1},\mu_{2},\ldots ,\mu_{2\nu })={\text{V}}^{-1}\text{A}\text{V}\\ \text{and}\;\;\mu_{2l} & ={\bar{\mu }}_{2l-1},\;l=1,\ldots ,\nu , \end{array}\}$$

where ***G***(***y***) are nonlinear coupling terms with *D****G***(**0**)=**0**. If, specifically, the *l*th linear mode of system (2.1) is brought to the standard form

$${\ddot{\eta }}_{l}+2\zeta_{l}\omega_{l}\dot{\eta_{l}}+\omega_{l}^{2}\eta_{l}=0,$$

with the damping ratio *ζ*_*l*_<1 and undamped natural frequency $\omega_{l}\in R$, then we obtain

$$\lambda_{l},{\bar{\lambda }}_{l}=-\zeta_{l}\omega_{l}\pm i\sqrt{1-\zeta_{l}^{2}}\omega_{l}$$

and hence the eigenvalues of ***A*** and ***Λ*** can be expressed as

5.3 $$\mu_{l},{\bar{\mu }}_{l}=e^{-\zeta_{l}\omega_{l}T}e^{\pm i\sqrt{1-\zeta_{l}^{2}}\omega_{l}T}.$$

We recall that if the sampling map ***F*** was constructed from observables along a two-dimensional invariant manifold *W* of system (2.2), then ***Λ*** has a complex conjugate pair of eigenvalues related to a pair of eigenvalues of A through the relationship (4.2). In particular, *W* is tangent to an underdamped modal subspace *E* of the equilibrium ***y***=**0** corresponding to the eigenvalue pair $\mu_{\ell },{\bar{\mu }}_{\ell }$ for some ℓ∈[1,2*n*−1], as implied by assumption (2.4).

The existence of a two-dimensional invariant manifold *W* tangent to a two-dimensional spectral subspace *E* of the linearized system (2.3) was first envisaged in the seminal work of Shaw & Pierre [4], and then extended to more general settings by the same authors and collaborators ([5–9] for reviews). On closer inspection, one finds that such invariant manifolds indeed exist under certain non-resonance conditions, but are non-unique and may have a low order of differentiability [10–12].

Following Haller & Ponsioen [12], we address this uniqueness and smoothness issue with the help of the following definition.

**Definition 5.1**

A *spectral submanifold* (SSM) W(E) corresponding to a spectral subspace E of the operator ***A*** is an invariant manifold of ***F*** with the following properties:

(i) W(E) is tangent to E at ***y***=0 and has the same dimension as E;
(ii) W(E) is strictly smoother than any other invariant manifold satisfying (i).

If it exists, an SSM serves as the unique nonlinear equivalent of the modal subspace E to the nonlinear system (2.2). By definition, all other invariant manifolds tangent to the same modal subspace have fewer derivatives, and hence any high-enough order Taylor expansion is only valid for the SSM.

Haller & Ponsioen [12] has pointed out that the more general and abstract results of Cabré *et al.* [16] imply the existence of SSMs for ***F*** under appropriate conditions on the spectrum of ***A***. Below, we recall these results stated specifically in the context of the sampling map ***F***. We note that by the conjugacy relation (4.1), the existence of a two-dimensional SSM for the sampling map ***F*** is equivalent to the existence of a two-dimensional SSM for the mechanical system (2.2), as long as the observable *φ* is generically chosen.

We start by considering a two-dimensional eigenspace $E\subset C^{2\nu }$ of the linearized sampling map ***A***, corresponding to the eigenvalue pair $\mu_{\ell },{\bar{\mu }}_{\ell }$. We define the *relative spectral quotient σ(E)*as the positive integer

5.4 $$\sigma (E)=\mathrm{Int}\left[\frac{\min_{j\neq \ell ,\ell +1}\log|\mu_{j}|}{\log|\mu_{\ell }|}\right]\in N^{+}.$$

For the linearized sampling map ***A*** (2.3), the constant σ(E) is the integer part of the ratio of the strongest decay rate towards the spectral subspace E to the decay rate along E. This integer ratio turns out to control the smoothness of the SSM W(E), as we shall see shortly.

We assume now that

5.5 σ(E)≤r,

i.e. the degree of smoothness of the sampling map ***F*** is at least as high as the relative spectral quotient of the modal subspace E. Finally, we assume that no low-order *external resonance* conditions hold between the eigenvalues $\mu_{\ell },{\bar{\mu }}_{\ell }$ inside E and the remaining eigenvalues of A outside E

5.6 $$\mu_{\ell }^{s_{1}}{\bar{\mu }}_{\ell }^{s_{2}}\neq \mu_{j},\;\forall \;j\neq \ell ,\ell +1,\;2\leq s_{1}+s_{2}\leq \sigma (E).$$

We then have the following existence and uniqueness result for SSMs of the sampling map.

**Theorem 5.2**

*Assume that conditions (*5.5*)–(*5.6*) are satisfied. Then, the following statements hold.*

(i) *There exists an SSM,* W(E), *for the nonlinear sampling map* ***F****, that is tangent to the invariant subspace* E *at the* ***ξ****=***0** *fixed point.*
(ii) *The invariant manifold* W(E) *is class C*^*r*^ *smooth and unique among all two-dimensional, class* $C^{\sigma (E)+1}$ *invariant manifolds of* ***F*** *that are tangent to* E *at* ***ξ****=***0**.
(iii) *The SSM* W(E) *can be viewed as a C*^*r*^ *immersion of an open set* $U\subset C^{2}$ *into the phase space* $C^{2\nu }$ *of* ***F*** *via a map
*
  5.7 $$\text{W}:U\subset C^{2}\to C^{2\nu },\;\text{W}(U)=W(E).$$
(iv) *There exists a C*^*r*^ *polynomial map* R:U→U *such that
*
  5.8 F∘W=W∘R,
  *i.e. the dynamics on the SSM, expressed in the coordinates* $\text{z}=(z_{\ell },{\bar{z}}_{\ell })\in U,$ *is given by the polynomial mapping* ***R****. This polynomial mapping only has terms up to order* $O(|\text{z}{|}^{\sigma (E)})$.
(v) *If, for some integer j*_0_*≥2, all internal non-resonance conditions
*
  5.9 $$\mu_{\ell }^{s_{1}}{\bar{\mu }}_{\ell }^{s_{2}}\neq \mu_{\ell },\mu_{\ell +1},\;j_{0}\leq s_{1}+s_{2}\leq \sigma (E)$$
  *holdwithin* E*, then the polynomial* ***R*** *in (*5.8*) can be selected to contain only terms up to order j*_0_*−1.*
(vi) *If the observable φ used in the construction of the sampling map* ***F*** *is generic, then a two-dimensional SSM, W(E), tangent to the subspace E at* ***x****=***0** *exists for the original system (*2.2*). The invariant manifold W(E) shares the properties (i)–(v) of* W(E) *due to the conjugacy relationship (*4.1*).*

**Proof.**

As explained in detail by Haller & Ponsioen [12], the proofs of statements (i)–(v) follow from a direct application of the more general Theorem 1.1 of Cabré *et al.* [16] on invariant manifolds tangent to spectral subspaces of arbitrary dimension, for mappings defined on Banach spaces. Statement (vi) can be concluded by invoking the strengthened version of Taken’s theorem [24,25] that we recalled in §4, and then interpreting the resulting structures of the Poincaré map ***Ξ***_T_ for the flow map of (2.2). □

**Remark 5.3**

Theorem 5.2 generally applies to any type of mechanical system of the form (2.1) and hence makes no specific assumption on the form or the magnitude of the damping, as we mention in the Introduction. If, however, the linearization of system (2.1) satisfies the classic proportional damping hypothesis, we can use (5.3) to rewrite the relative spectral quotient defined in (5.4) as

5.10 $$\sigma (E)=\mathrm{Int}\left[\frac{\max_{j\neq \ell ,\ell +1}\zeta_{j}\omega_{j}}{\zeta_{\ell }\omega_{\ell }}\right].$$

In this case, the expressions in the external non-resonance conditions (5.6) for E take the specific form

$$\left(\begin{array}{c} (s_{1}+s_{2})\zeta_{\ell }\omega_{\ell }\\ (s_{1}-s_{2})\omega_{\ell }\sqrt{1-\zeta_{\ell }^{2}}\;\;\mathrm{mod}\;\;\;\frac{2\pi }{T} \end{array}\right)\neq \left(\begin{array}{c} \zeta_{j}\omega_{j}\\ \omega_{nj}\sqrt{1-\zeta_{j}^{2}}\;\;\mathrm{mod}\;\;\;\frac{2\pi }{T} \end{array}\right),\;2\leq s_{1}+s_{2}\leq \sigma (E),$$

where mod denotes the modulo operation that takes sampling into account. In the limit of zero damping, an external resonance for E means that a frequency *ω*_*j*_ outside E is an integer multiple of the frequency *ω*_ℓ_ inside E.

Statements (iii)–(v) of theorem 5.2 imply that, unlike in the Shaw & Pierre [4] construction, the SSM inferred from the results of Cabré *et al.* [16] is not assumed to be a graph over the subspace E in the phase space of ***F***. This allows W(E) to be constructed on larger domains on which it can produce folds over E. This parametrization approach to SSM construction was also rediscovered recently by Cirillo *et al.* [11] under the assumption that the flow is analytically linearizable near the fixed point ***x***=**0**. Analytic linearization does not allow for any resonance in the spectrum of ***A*** and, in return, transforms the full dynamics of the mapping ***F*** into that of ***Λy***. In the case of a near-resonance—which arises for all weakly underdamped modes, as we shall see below—analytic linearization can therefore only be constructed on a very small domain near the fixed point. This disallows the type of direct identification of nonlinear terms that we discuss next.

## 6. Dynamics on spectral submanifolds: backbone curves

Since |*μ*_ℓ_|<1 holds by assumption (2.4), we find that, strictly speaking, the internal non-resonance condition (5.9) is always satisfied for non-zero damping.

As seen in the construct of Cabré *et al.* [16], however, even an approximate resonance $\mu_{\ell }^{s_{1}}{\bar{\mu }}_{\ell }^{s_{2}}\approx \mu_{j}$ causes the near-identity transformation $\left(\xi_{\ell },{\bar{\xi }}_{\ell })\mapsto (z_{\ell },{\bar{z}}_{\ell }\right)$ to have small denominators, limiting the existence of this transformation to a tiny neighbourhood of the ***ξ***=**0** fixed point. Since our interest here is to obtain an approximation of the dynamics of ***F*** on a sizeable neighbourhood of the fixed point within the SSM, we do not insist on the removal of approximately resonant terms in the $\left(z_{\ell },{\bar{z}}_{\ell }\right)$ coordinate system. Rather, we observe that, for small damping ratios (i.e. for |*μ*_ℓ_|≈1), the low-order near-resonance relationships

6.1 $$\mu_{\ell }^{2}{\bar{\mu }}_{\ell }\approx \mu_{\ell },\;\mu_{\ell }{\bar{\mu }}_{\ell }^{2}\approx {\bar{\mu }}_{\ell }$$

are always satisfied, and hence the minimal possible integer *j*_0_ satisfying (6.2) (with ≠ replaced with ≉) is *j*_0_=1. Accordingly, the approximately failing resonance conditions in (5.9) prompt us to seek ***R*** (cf. statement (iv) of theorem 5.2) as a cubic polynomial of the form

6.2 $$\text{R}(\text{z})=\left(\begin{array}{c} \mu_{\ell }z_{\ell }+\beta_{\ell }z_{\ell }^{2}{\bar{z}}_{\ell }+\cdots \\ {\bar{\mu }}_{\ell }{\bar{z}}_{\ell }+\bar{\beta_{\ell }}z_{\ell }{\bar{z}}_{\ell }^{2}+\cdots \end{array}\right).$$

Introducing polar coordinates *z*=*ρ* *e*^*iθ*^, we can further transform (6.2) to the real amplitude–phase components

6.3 $$\rho_{\ell }\mapsto \rho_{\ell }|\mu_{\ell }+\beta_{\ell }\rho_{\ell }^{2}|$$

and

6.4 $$\theta_{\ell }\mapsto \theta +\arg(\mu_{\ell }+\beta_{\ell }\rho_{\ell }^{2}).$$

Equation (6.4) then provides an instantaneous frequency of nonlinear oscillations, with the instantaneous oscillation amplitude governed by equation (6.3). Given that the sampling period we have used is *T*, the leading-order approximation of the instantaneous oscillation frequency in the original nonlinear system (2.2) is

6.5 $$\omega (\rho_{\ell })=\frac{\arg(\mu_{\ell }+\beta_{\ell }\rho_{\ell }^{2})}{T}.$$

We take the instantaneous leading-order amplitude of the corresponding trajectories of (2.2) to be the norm of $\text{z}(\rho_{\ell },\theta_{\ell })=(\rho_{\ell }\;e^{i\theta_{\ell }},\rho_{\ell }\;e^{-i\theta_{\ell }})$ in the original ***ξ*** coordinates. A nominal instantaneous amplitude *Amp*(*ρ*) of the vibration can then be calculated from (5.1) as the *L*_2_-norm of the norm of ***z***(*ρ*_ℓ_,*θ*_ℓ_) in the original ***ξ*** coordinates

6.6 $$\mathrm{Amp}(\rho_{\ell })=\sqrt{\frac{1}{2\pi }\int_{0}^{2\pi }|\text{V}\text{W}(\text{z}(\rho_{\ell },\theta_{\ell })){|}^{2}\;d\theta .}$$

Here, the linear map ***V*** is the one appearing in (5.1), and the mapping ***W*** in the one appearing in (5.7).

**Definition 6.1**

We call the parametrized curve

6.7 $$B_{\ell }={\left\{\omega (\rho_{\ell }),\;\mathrm{Amp}(\rho_{\ell })\right\}}_{\rho_{\ell }\in R^{+}}\subset R^{2}$$

the *backbone curve*associated with the nonlinear dynamics on the SSM, W(E).

The key to the computation of the backbone curve (6.7) is, therefore, the computation of the single complex coefficient *β*_ℓ_ and of the mapping ***W***(***z***). This is because both the eigenvalue *μ*_ℓ_ and the sampling time *T* are already assumed to be known.

**Remark 6.2**

It is often desirable to translate the *φ*-based backbone curve $B_{\ell }$ defined in (6.7) to a backbone curve observed directly for a given mechanical coordinate *q*_*j*_. When the observable is an invertible function of such a *q*_*j*_, that is, $\varphi (\text{q},\dot{\text{q}})=\varphi (q_{j})$, we can use the inverse, defined by $q_{j}=P(\xi )=P(\varphi (q_{j}))$. Also note that, by the definition of the observable space, coordinates of ***ξ*** are just sampled values of the same observed quantity. Therefore, when calculating an amplitude, it is reasonable to consider just a single component of ***ξ***, for example, *ξ*_1_. With this in mind, we consider P functions in the particular form $P(\xi )=P(\xi_{1})$. As a result, the observed amplitude in the *q*_*j*_ mechanical coordinate can be computed as

$$\mathrm{Amp}(\rho_{\ell })=\sqrt{\frac{1}{2\pi }\int_{0}^{2\pi }|P(\text{V}\text{W}(\text{z}(\rho_{\ell },\theta_{\ell }))){|}^{2}\;d\theta }.$$

To compute the complex parameter *β* in equation (6.5), we need to solve (5.8). To this end, we seek the Taylor series coefficients of the *j*th coordinate functions, $W_{j}(\text{z})\in C$, *j*=1,…,2*ν*, of the mapping ***W***(***z***) up to third order. Similarly, we seek the third-order Taylor coefficient $\beta_{\ell }\in C$ of the polynomial mapping ***R***(***z***) defined in (6.2). All these unknowns should be expressed in the end as functions of the *j*th coordinate functions $G_{j}(\text{y})\in C,$ *j*=1,…,2*ν*, of the nonlinear part ***G***(***y***) of the transformed sampling map ***F***. The relevant Taylor expansions are in the general form

6.8 $$G_{j}(\text{y})=\sum_{|\text{m}|\geq 2}g_{j}^{\text{m}}{\text{y}}^{\text{m}},\;\text{m}\in N^{2\nu },\;g_{j}^{\text{m}}\in C,\;j=1,\ldots ,2\nu$$

and

6.9 $$W_{j}(\text{z})=\sum_{|\text{s}|\geq 1}w_{j}^{\text{s}}{\text{z}}^{\text{s}},\;\text{s}\in N^{2},\;w_{j}^{\text{s}}\in C,\;j=1,\ldots ,2\nu .$$

In expressing the solutions of (5.8) in terms of these coefficients, we will use the shorthand notation (*p*@*j*) for an integer multi-index whose elements are zero, except for the one at the *j*th position, which is equal to *p*

$$(p@j):=\left(0,\ldots ,\underset{j-1}{0},\underset{j}{p},\underset{j+1}{0},\ldots ,0\right)\in N^{2\nu }.$$

We will also concatenate this notation to refer to multi-indices whose entries are zero except at prescribed locations

$$(p@j_{1},q@j_{2}):=\left(\underset{}{0},\ldots ,\underset{j_{1}-1}{0},\underset{j_{1}}{p},\underset{j_{1}+1}{0},\underset{\cdots }{\ldots },\underset{j_{2}-1}{0},\underset{j_{2}}{q},\underset{j_{2}+1}{0},\underset{\cdots }{\ldots },\underset{}{0}\right)\in N^{2\nu }.$$

For *j*_1_≡*j*_2_=*j*, we let

$$(p@j,q@j):=((p+q)@j)=\left(\ldots ,\underset{j-1}{0},\underset{j}{p+q},\underset{j+1}{0},\underset{\cdots }{\ldots }\right)\in N^{2\nu }.$$

With all this notation, we obtain the following result.

**Theorem 6.3**

*Suppose that the assumptions of theorem *5.2 *hold but with the strengthened version
*

6.10 $$\mu_{\ell }^{s_{1}}{\bar{\mu }}_{\ell }^{s_{2}}≉\mu_{j},\;\forall \;j\neq \ell ,\ell +1,\;1\leq s_{1}+s_{2}\leq \sigma (E)$$

*of the external non-resonance condition (*5.6*). Then, for any j∈[1,2ν], the jth coordinate function W*_*j*_ *of the mapping* ***W*** *and the cubic Taylor coefficient β*_ℓ_ *of the conjugate map* ***R*** *are given by the following formulae:
*

$$\begin{array}{rl} w_{j}^{\left(1,0\right)} & =\delta_{j\ell },\;w_{j}^{\left(0,1\right)}=\delta_{j(\ell +1)},\\ w_{j}^{\left(2,0\right)} & =\frac{g_{j}^{\left(2@\ell \right)}}{\mu_{\ell }^{2}-\mu_{j}},\;w_{j}^{\left(1,1\right)}=\frac{g_{j}^{\left(1@\ell ,1@(\ell +1)\right)}}{\mu_{\ell }{\bar{\mu }}_{\ell }-\mu_{j}},\;w_{j}^{\left(0,2\right)}=\frac{g_{j}^{\left(2@(\ell +1)\right)}}{{\bar{\mu }}_{\ell }^{2}-\mu_{j}},\\ w_{j}^{\left(3,0\right)} & =\frac{\sum_{q=1}^{2\nu }(1+\delta_{\ell q})g_{j}^{\left(1@\ell ,1@q\right)}w_{q}^{\left(2,0\right)}+g_{j}^{\left(3@\ell \right)}}{\mu_{\ell }^{3}-\mu_{j}},\\ w_{j}^{\left(0,3\right)} & =\frac{\sum_{q=1}^{2\nu }(1+\delta_{(\ell +1)q})g_{j}^{\left(1@(\ell +1),1@q\right)}w_{q}^{\left(0,2\right)}+g_{j}^{\left(3@(\ell +1)\right)}}{{\bar{\mu }}_{\ell }^{3}-\mu_{j}}. \end{array}$$

*and
*

$$\begin{array}{rl} w_{j}^{\left(2,1\right)} & =(1-\delta_{j\ell })\\ & \;\times \frac{\sum_{q=1}^{2\nu }[(1+\delta_{\ell q})g_{j}^{\left(1@\ell ,1@q\right)}w_{q}^{\left(1,1\right)}+(1+\delta_{(\ell +1)q})g_{j}^{\left(1@(\ell +1),1@q\right)}w_{q}^{\left(2,0\right)}]+g_{j}^{\left(2@\ell ,1@(\ell +1)\right)}}{\mu_{\ell }^{2}{\bar{\mu }}_{\ell }-\mu_{j}},\\ w_{j}^{\left(1,2\right)} & =(1-\delta_{j(\ell +1)})\\ & \;\times \frac{\sum_{q=1}^{2\nu }[(1+\delta_{\ell q})g_{j}^{\left(1@\ell ,1@q\right)}w_{q}^{\left(0,2\right)}+(1+\delta_{(\ell +1)q})g_{j}^{\left(1@(\ell +1),1@q\right)}w_{q}^{\left(1,1\right)}]+g_{j}^{\left(2@(\ell +1),1@\ell \right)}}{\mu_{\ell }{\bar{\mu }}_{\ell }^{2}-\mu_{j}},\\ \beta_{\ell } & =\sum_{q=1}^{2\nu }[(1+\delta_{\ell q})g_{\ell }^{\left(1@\ell ,1@q\right)}w_{q}^{\left(1,1\right)}+(1+\delta_{(\ell +1)q})g_{\ell }^{\left(1@(\ell +1),1@q\right)}w_{q}^{\left(2,0\right)}]+g_{\ell }^{\left(2@\ell ,1@(\ell +1)\right)}. \end{array}$$

**Proof.**

For the proof, see the electronic supplementary material, section A. □

**Remark 6.4**

Theorem 6.3 only provides the solution of the homological equation (5.8) up to cubic order. This equation, however, can be solved by symbolic computations up to any order for the Taylor coefficients of the functions ***W*** and ***R***. For instance, up to quintic order, the near-resonance conditions (6.1) imply the general form

$$\text{R}(\text{z})=\left(\begin{array}{c} \mu_{\ell }z_{\ell }+\beta_{\ell }z_{\ell }^{2}{\bar{z}}_{\ell }+\gamma_{\ell }z_{\ell }^{3}{\bar{z}}_{\ell }^{2}+\cdots \\ {\bar{\mu }}_{\ell }\bar{z_{\ell }}+\bar{\beta_{\ell }}z_{\ell }{\bar{z}}_{\ell }^{2}+{\bar{\gamma }}_{\ell }z_{\ell }^{2}{\bar{z}}_{\ell }^{3}+\cdots \end{array}\right)$$

for the polynomial conjugate dynamics on the SSM E. The coefficient *γ*_ℓ_ as well as the quartic and quintic terms of ***W*** can be found recursively from equation (5.8), following the procedure outlined in the electronic supplementary material, section A. The sampling map restricted to the SSM W(E) can be written in polar coordinates up to quintic order as

6.11 $$\rho_{\ell }\mapsto \rho_{\ell }|\mu_{\ell }+\beta_{\ell }\rho_{\ell }^{2}+\gamma_{\ell }\rho_{\ell }^{4}|$$

and

6.12 $$\theta_{\ell }\mapsto \theta +\arg(\mu_{\ell }+\beta_{\ell }\rho_{\ell }^{2}+\gamma_{\ell }\rho_{\ell }^{4}),$$

yielding the instantaneous oscillation frequency in the original nonlinear system (2.2) as

6.13 $$\omega (\rho_{\ell })=\frac{\arg(\mu_{\ell }+\beta_{\ell }\rho_{\ell }^{2}+\gamma_{\ell }\rho_{\ell }^{4})}{T}.$$

The formulae (6.6) and (6.13) then give a refined, quintic approximation for the backbone curve $B_{\ell }$. The same procedure applies to further, higher-order approximations of $B_{\ell }$.

**Remark 6.5**

The external non-resonance condition (5.6) of theorem 6.3 only excludes quadratic and higher-order resonances. As a result, for overdamped SSMs E with eigenvalues $\mu_{\ell },\mu_{\ell +1}\in R$, condition (5.6) would still technically allow for a 1:1 external resonance (characterized by *s*_1_=1 and *s*_2_=0) with an eigenvalue $\mu_{j}\in R$ outside E . In our setting, however, the damping is assumed weak and hence an approximate 1:1 external resonance *μ*_ℓ_≈*μ*_*j*_ implies an approximate external 2:1 resonance $\mu_{\ell }^{2}\mu_{\ell +1}^{1}\approx \mu_{j}$, resulting in small denominators for $w_{j}^{\left(1,2\right)}$ and $w_{j}^{\left(2,1\right)}$ in the statement of theorem 6.3. The strengthened non-resonance condition (6.10) serves to exclude this case, as well as other cases of near-resonance that create non-zero but small denominators for the coefficients in theorem 6.3. Although technically non-zero, such small denominators are undesirable as they may significantly decrease the phase space domain on which the formulae of the theorem give a good approximation for the underlying SSM and its reduced dynamics. One may improve this approximation by adding the resonant mode with frequency *ω*_*j*_ to the original spectral subspace E, whose dimension then becomes four. The general results described by Haller & Ponsioen [12] can then be used to derive expressions for the corresponding enlarged (four-dimensional) SSM W(E) and the reduced dynamics it carries.

## 7. Reconstruction of the sampling map from data

In an experimental setting, backbone-curve identification via theorem 6.3 requires the fitting of a model of ***F*** to observations using an appropriate set of basis functions. Owing to the polynomial form (3.5) of ***F***, the required basis functions are precisely vector-valued monomials of the variables *ξ*_1_,…,*ξ*_2*ν*_ not including constant terms. The lack of constant terms follows from the assumption (3.4), which can always be satisfied by an appropriate shift of coordinates, if necessary.

For the polynomial-based model-identification for ***F***, we employ a nonlinear autoregressive model (NAR) [26]. We order all integer vectors ***m*** up to order |***m***|=*r* (i.e. all index vectors in the leading-order Taylor expansion (3.5)) into a series {***m***^*l*^} so that

$${\text{m}}^{v}≺{\text{m}}^{w}\;⟺\;m_{j}^{v}\leq m_{j}^{w},\;j=1,\ldots ,2\nu .$$

We can then write the yet unknown, *r*th-order Taylor expansion of ***F*** in the compact form

7.1 $$\text{F}(\xi )=\text{K}\psi (\xi )+\text{r}(\xi ),\;\psi_{l}(\xi )=\xi^{{\text{m}}^{l}},$$

where $\text{K}\in R^{2\nu \times N}$ is a rectangular matrix, to be determined by minimizing the residual term $\text{r}(\xi )\in R^{2\nu }$ on assimilated data in the ℓ^2^-norm.

The input data to be assimilated into the NAR model consists of *P* sequences of *M*_*p*_>2*ν*-long observations, ${\left\{\xi_{k}^{p}\right\}}_{k=0}^{M_{p}-2\nu }$ , *p*=1,…,*P*, with each observation sequence ${\left\{\xi_{k}^{p}\right\}}_{k=0}^{M_{p}-2\nu }$ defined as in (3.1). The ℓ^2^-norm of ***r***(***ξ***) on ${\left\{\xi_{k}^{p}\right\}}_{k=0}^{M_{p}-2\nu }$ over all *P* observation sequences is then given by

$$\mathrm{Err}=\sum_{p=1}^{P}\sum_{k=0}^{M_{p}-2\nu }|\text{r}(\xi_{k}^{p}){|}^{2}=\sum_{p=1}^{P}\sum_{k=0}^{M_{p}-2\nu }|\text{K}\psi (\xi_{k}^{p})-\xi_{k+1}^{p}{|}^{2}.$$

The matrix ***K*** that minimizes this norm is obtained by solving the equation *dErr*/*d****K***=**0** for ***K***. This classic computation yields ***K***=***QP***^−1^, where

$$\text{P}=\sum_{p=1}^{P}M_{p}^{-1}\sum_{k=0}^{M_{p}-2\nu }\psi (\xi_{k}^{p})\psi^{⋆}(\xi_{k}^{p})$$

and

$$\text{Q}=\sum_{p=1}^{P}M_{p}^{-1}\sum_{k=0}^{M_{p}-2\nu }\xi_{k+1}^{p}\psi^{⋆}(\xi_{k}^{p})$$

with ⋆ denoting the transposition. With this notation, the reconstructed nonlinear sampling map is

7.2 $$\tilde{\text{F}}(\xi )=\text{Q}{\text{P}}^{-1}\psi (\xi ),$$

which we will use instead of the exact sampling map ***F*** in our analysis.

Assimilating multiple measurement sequences (i.e. using *P*>1) generally reduces the effect of zero-mean additive noise on the model reconstruction. More importantly, using measurements from vibrations decaying near *P* natural frequencies of interest allows us to build a single reduced-order discrete model map $\tilde{\text{F}}$ that simultaneously captures nonlinear behaviour near all these natural frequencies. The choice of the ℓ^2^ optimization above was mostly dictated by convenience; in some situations, minimization of ***r***(***ξ***) in the ℓ^1^ or $\ell^{\infty }$ norms might be more beneficial.

Since we do not know the invariant manifold W(E) exactly, we will construct (7.2) from observed nonlinear vibration decay measurements initiated along two-dimensional modal subspaces of *D****Ξ***_T_(0). In practice, these subspaces can be approximated from linear modal analysis.

## 8. Summary of spectral submanifold-based backbone-curve identification algorithm

We now briefly summarize the steps in the approach, we have developed in the preceding sections:

1. Fix a generic scalar observable $\varphi (\text{q},\dot{\text{q}})$ and a sampling time *T*>0 for the mechanical system (2.1). Also fix an integer *ν*≥3 as the number of SSMs to be identified for system (2.1). Finally, select an integer r=max|m| for the maximum degree of the polynomials used in the construction of the NAR model (7.1) for the sampling map $\tilde{\text{F}}(\xi )$ with $\xi \in R^{2\nu }$.
2. Collect *P* sequences of *M*_*p*_-long observations, ${\left\{\xi_{k}^{p}\right\}}_{k=0}^{M_{p}-2\nu }$, by letting
  $$\begin{array}{rl} \xi_{k}^{p} & =(\varphi (\text{q}(kT),\dot{\text{q}}(kT)),\ldots ,\varphi (\text{q}((k+2\nu -1)T),\dot{\text{q}}((k+2\nu -1)T))),\\ & \;p=1,\ldots ,P,\;k=0,\ldots ,M_{p}-2\nu . \end{array}$$
3. Compute the approximate 2*ν*-dimensional sampling map $\tilde{\text{F}}(\xi )$ from formula (7.2).
4. Transform $\tilde{\text{F}}(\xi )$ to its complex diagonal form (5.2).
5. Using theorem 5.2, compute the leading-order Taylor coefficients of the mapping ***W***(***z***_ℓ_) and the leading-order polynomial coefficient *β*_ℓ_ for each SSM, W(E), provided that the non-resonance condition (5.6) holds.
6. Calculate the backbone curve $B_{\ell }$ defined in (6.7) for W(E). Higher-order approximations to $B_{\ell }$ can be computed similarly, as summarized briefly in remark 6.4.

This algorithm provides the simplest possible first-order approach to SSM-based backbone curve reconstruction. This simplest approach does not fully exploit the uniqueness class $C^{\sigma (E)+1}$ of W(E), as guaranteed by theorem 5.2. To obtain higher precision approximations to $B_{\ell }$, one must derive higher-order Taylor coefficients of ***W***(***z***_ℓ_) and *β*_ℓ_ from the invariance condition (5.8), which we do not pursue here.

## 9. Examples

We now demonstrate the application of SSM-based model reduction and backbone-curve reconstruction in two examples. First, we consider a two-degree-of-freedom damped, nonlinear oscillator model to benchmark data-based SSM reconstruction in a case where analytic, model-based computations are also possible. Second, we use vibration decay data from an oscillating beam experiment to illustrate the direct computation of backbone curves $B_{\ell }$ from an experimentally reconstructed sampling map $\tilde{\text{F}}.$

### (a) Modified Shaw–Pierre example

We slightly modify here the two-degree-of-freedom oscillator studied by Shaw & Pierre [4] by making the damping matrix proportional to the stiffness matrix in the linearized problem. The first-order equations of motion we study are

9.1 $$\begin{array}{rl} {\dot{x}}_{1} & =v_{1},\\ {\dot{x}}_{2} & =v_{2},\\ {\dot{v}}_{1} & =-cv_{1}-k_{0}x_{1}-\kappa x_{1}^{3}-k_{0}(x_{1}-x_{2})-c(v_{1}-v_{2})\\ \text{and}\;\;{\dot{v}}_{2} & =-cv_{2}-k_{0}x_{2}-k_{0}(x_{2}-x_{1})-c(v_{2}-v_{1}). \end{array}\}$$

We first calculate SSMs and backbone curves for this system using a formulation for continuous dynamical systems, as described in the electronic supplementary material, section B. We then emulate an experimental sampling of the vibrations of system (9.1) and reconstruct SSMs and backbone curves from the sampled data using the discrete methodology described in §§3–7.

System (9.1) is analytic, hence we have *r*=*a* in our notation. The natural frequencies and damping ratios are

$$\omega_{1}=\sqrt{k_{0}},\;\omega_{2}=\sqrt{3k_{0}},\;\zeta_{1}=\frac{c}{2\sqrt{k_{0}}}\;\mathrm{and}\;\zeta_{2}=\frac{\sqrt{3}c}{2\sqrt{k_{0}}},$$

yielding the complex eigenvalues

$$\lambda_{1,2}=-\frac{c}{2}\pm i\sqrt{k_{0}\left(1-\frac{c^{2}}{4k_{0}}\right)}\;\mathrm{and}\;\lambda_{3,4}=-\frac{3c}{2}\pm i\sqrt{3k_{0}\left(1-\frac{3c^{2}}{4k_{0}}\right)},$$

where we have assumed that both modes are underdamped, i.e. $c<2\sqrt{k_{0}/3}.$

For the corresponding two-dimensional modal subspaces *E*_1_ and *E*_2_, remark 5.3 gives

σ(E1)=Int[Re λ3Re λ1]=Int[(3c/2k0)3k0(c/2k0)k0]=3

and

σ(E2)=Int [Re λ1Re λ3]=Int [(c/2k0)k0(3c/2k0)3k0]=0.

Therefore, in electronic supplementary material, section B, theorem 1, there exist two-dimensional, analytic SSMs, *W*(*E*_1_) and *W*(*E*_2_) that are unique among *C*^4^ and *C*^1^ invariant manifolds tangent to *E*_1_ and *E*_2_, respectively, at the origin.

By the analytic calculations detailed in the electronic supplementary material, section C, we obtain the corresponding backbone-curve parametrizations

$$\omega (\rho_{1})=\frac{1}{2}\left(\sqrt{4k_{0}-c^{2}}+\frac{3\kappa }{\sqrt{4k_{0}-c^{2}}}\rho_{1}^{2}\right),\;\mathrm{Amp}(\rho_{1})\approx 2\rho_{1}$$

and

$$\omega (\rho_{2})=\frac{1}{2}\left(\sqrt{3(4k_{0}-3c^{2})}+\frac{\sqrt{3}\kappa }{\sqrt{4k_{0}-3c^{2}}}\rho_{2}^{2}\right),\;\mathrm{Amp}(\rho_{2})\approx 2\rho_{2}.$$

To determine these backbone curves for the damping and stiffness values *c*=0.003, *k*_0_=1 and *κ*=0.5, we emulate a hammer experiment that gives an initial condition in the modal subspaces *E*_1_ and *E*_2_ to the full nonlinear system. The precise initial conditions of the two decaying signals are

9.2 $${\text{x}}^{\left(1\right)}(0)=\frac{1}{\sqrt{3}}{\left(2,2,0,0\right)}^{T}\in E_{1},{\text{x}}^{\left(2\right)}(0)=\frac{1}{3}{\left(-2,2,0,0\right)}^{T}\in E_{2}.$$

We sample the solutions starting from these points 8000 times with the sampling interval *T*=0.8. In terms of our notation, we, therefore, have *P*=2, *M*_1_=*M*_2_=8000. As observable, we choose the velocity of the first mass was i.e. let *φ*(***x***)=*v*_1_, to emulate an experimental procedure that renders only velocities (as in our second example below). As the minimal embedding dimension for the sampling map $\tilde{\text{F}}(\xi )$, Step 1 of the algorithm in §8 gives 2*ν*=6. In the present example, however, we know that *E*_1_ and *E*_2_ are properly embedded already in the four-dimensional system (9.1), and hence we select 2*ν*=4 instead.

**Figure 2.**
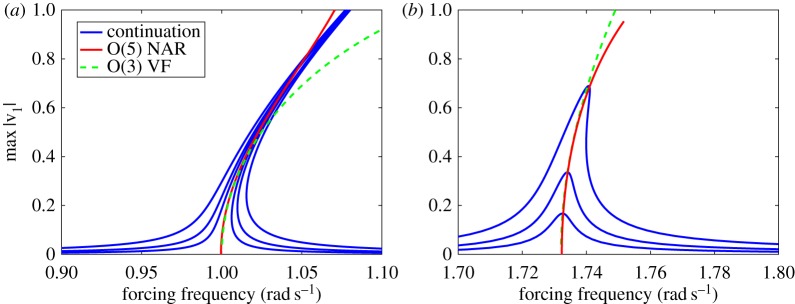
Backbone curves and forced response of the mechanical system (9.1) for the first (*a*) and second (*b*) natural frequency. Blue curves show forced responses of the lightly damped system *c*=0.0005. Red continuous lines show the fifth-order backbone curves recovered from our algorithm by sampling two freely decaying trajectories with initial conditions (9.2). Green dashed lines show the O(3) analytic calculation of the same backbone curves using the electronic supplementary material, Theorem S2. (Online version in colour.)

The red curve in figure 2 shows a closed-form quintic computation (cf. remark 6.4) of the backbone curves $B_{1}$ and $B_{2}$ from the data-assimilating discrete algorithm described in §8. The two trajectories used as inputs for this algorithm were launched from the initial conditions (9.2).

For comparison, the green dashed line in the same figure shows a cubic analytic computation of the backbone curves based on the continuous-time (vector-field) formulation we have given in the electronic supplementary material, section B, theorem 2. Finally, we have used numerical continuation [27] at various amplitudes of forcing to find periodic orbits for low damping with *c*=0.0005. The resulting periodic response amplitudes are shown in figure 2 in blue as functions of the forcing frequency. The O(5) backbone curve fits remarkably well with the peaks of the blue curves, especially considering that these backbone curves were computed from just two sampled trajectories. The robustness of the backbone curves is also noteworthy, given that the blue curves were obtained for substantially lower damping values.

### (b) Clamped–clamped beam

**Figure 3.**
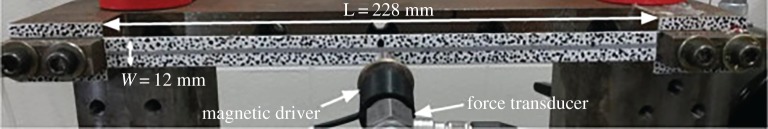
The experimental set-up for constructing backbone curves for a clamped–clamped beam. Adapted from [21]. (Online version in colour.)

We now test the trajectory-data-assimilating backbone-curve reconstruction algorithm of §8 on experimental data obtained from the vibration tests described in [21]. We show the experimental device, a beam clamped at both ends, in figure 3.

The data comprise freely decaying velocity signals measured at the midpoint of the beam with initial conditions selected near three assumed SSMs. These initial conditions were obtained experimentally by force appropriation (cf. the Introduction). The decaying signals were initialized at maximal response amplitudes obtained from single-frequency force appropriation. Three signals were assimilated, corresponding to each natural frequency, which gives *P*=3 in our notation. Each signal was resampled with time period *T*=0.97656 *ms*. The lengths of the three signals were *M*_1_=3892, *M*_2_=2458 and *M*_3_=1055 samples.

The second mode was not analysed in [21], because the node of this mode is precisely at the midpoint of the beam, which can significantly deteriorate measurement accuracy. We list the natural frequencies identified from the NAR model in table 1. In the last row of the same table, we also show the spectral quotients obtained from formula (5.10) for the three modes.

**Table 1.** Natural frequencies and damping ratios for the first three modes of the clamped–clamped beam as determined by our algorithm. Also shown are the spectral quotients $\sigma (E_{l})$. The *ω*_*l*_ values are close to those linearly identified in [21], but the *ζ*_*l*_ values are markedly different. The nonlinear model identification used here does not need to capture these linearized parameter values with the same accuracy as a purely linear analysis would. We only present the nonlinear identification results here for completeness.

mode	*l*=1	*l*=2	*l*=3
*ω*_*l*_ (Hz)	47.4921	167.1512	368.4577
*ζ*_*l*_	0.1833	0.0183	0.0019
$\sigma (E_{l})$	0	2	12

Based on table 1, theorem 5.2 gives a unique SSM $W(E_{1})$ within the class of *C*^1^ manifolds. This is because the first mode represents the fastest decaying linear subspace of oscillations, admitting a unique nonlinear continuation in the form of the fast SSM $W(E_{1})$. The second (slow) SSM $W(E_{2})$ and the third (intermediate) SSM $W(E_{3})$ are only unique among *C*^3^ and *C*^13^ invariant manifolds tangent to the spectral subspaces $E_{2}$ and $E_{3}$, respectively. This suggests that backbone reconstruction techniques that do not consider the smoothness of the underlying SSM are expected to show greater uncertainty for the second and the third mode.

We seek to obtain an NAR model for the delay embedding of all three modes in table 1. This means we have *ν*=3, and hence the required minimal dimension of the reconstructed nonlinear sampling map $\tilde{\text{F}}(\xi )$ is 2*ν*=6. We employ a third-order polynomial model (*r*=3) in the NAR model of §7. Accordingly, we construct the dynamics on the three SSMs up to cubic order (cf. formula (6.2)), with the Taylor coefficients of ***W*** and ***R*** computed from the formulae given in theorem 6.3.

**Figure 4.**
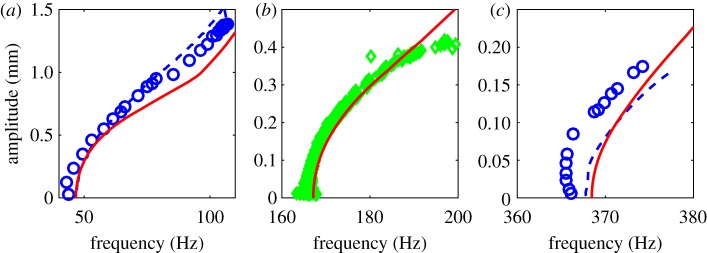
Backbone curves for the first (*a*), second (*b*) and third (*c*) natural frequencies of a clamped–clamped beam. Solid lines: backbone curves computed from a data-assimilating cubic-order SSM reduction, as summarized in §8; Dashed lines: backbone curves obtained from individual decaying signals using a Hilbert transform approach [28]. Circles: force-appropriation results using stepped sine forcing. Diamonds: Instantaneous amplitude–frequency curves inferred from decaying vibration data by calculating zero crossings of the signal to estimate vibration period. Apart from the solid lines, all data were obtained directly from the experiments of Ehrhardt & Allen [21]. (Online version in colour.)

Figure 4 shows the results of our computations. To be consistent with Ehrhardt & Allen [21], we compute the response amplitudes by dividing the available instantaneous velocity amplitudes with their corresponding instantaneous frequencies. This simple devision, therefore, represents the function P from the observable space to the relevant coordinate space (cf. remark 6.2). The resulting backbone curve of the first SSM matches well previous results. This is expected, because this SSM is the most robust among the three SSMs considered here (unique already among *C*^1^ invariant manifolds tangent to the spectral subspace $E_{1}$). The kink at about 90 Hz appears to be an artefact of O(3) model fitting. Higher-amplitude results for this SSM (not shown) are even less reliable because of the relative sparsity of the data there.

There is no comparison available from Ehrhardt & Allen [21] for the second backbone curve, but the backbone curve we compute for this case is consistent with the instantaneous amplitude–frequency data (green) inferred from decaying vibrations.

For the third SSM, there is a noticeable offset between the force appropriation result and the rest of the curves. Our calculations, however, match closely the instantaneous amplitude–frequency data, with the backbone curve obtained from resonance decay. Capturing the SSM corresponding to this mode uniquely would theoretically require a high-order, O(13) approximation. This, however, would be unfeasible given the limited amount of data available.

## 10. Discussion

We have developed a method to extract two-dimensional SSMs and their associated backbone curves for multi-degree-of-freedom nonlinear mechanical vibrations. We computed the SSMs explicitly as two-dimensional invariant manifolds of a low-order, discrete model system fitted to sampled trajectory data. Restricted to the SSMs, this model is guaranteed to be conjugate to the full mechanical system by the classic Takens embedding theorem, as long as the data assimilated into the model is from a generic observable.

We have illustrated the power of this approach by calculating backbone curves of the reconstructed dynamics on the SSMs in two examples. In our first example, a two-degree-of-freedom analytic model, we verified the trajectory-data-based backbone-curve computation via an analytic calculation of the same curve for the full, continuous-time system, as well as by numerical continuation. In our second example, we compared the data-assimilated construction of the backbone curves with various experimentally inferred curves and found close agreement.

To obtain SSMs and their reduced dynamics analytically, we use the parametrization method in [16], which is generally not limited to a small neighbourhood of a fixed point. In addition, the parametrization method allows for the presence of resonances or near-resonances that unavoidably arise in underdamped oscillations (cf. equation (6.1)). This is in contrast with parametrized SSM constructions based on Sternberg’s analytic linearization theorem (cf. [11]) that exclude any resonance in the linearized spectrum. When applied in the near-resonant case, the domain of validity of the analytic linearization and the manifolds construction is, therefore, exceedingly small. In addition, reliance on analytic linearization excludes the possibility of extracting backbone curves, which arise from the nonlinear dynamics on the reconstructed SSM.

The parametrization method enables us to identify SSMs with high accuracy on larger domains, even from relatively low-amplitude trajectory samples, as long as we use high-enough order in the approximations for the SSMs and its reduced dynamics. This high-enough order ensures the accurate interrogation of nonlinearities even from low-amplitude signals. In our examples, a fifth-order computation yielded remarkably accurate results even for higher-amplitude ranges of the backbone curve, while a third-order computation was effective for lower-amplitude backbone-curve ranges.

Our algorithm is designed so that an arbitrary number of decaying vibrations can be assimilated into the underlying reduced-order discrete NAR model. Unlike normal forms derived specifically for given modes of interest, our model incorporates the dynamics of all modes of interest simultaneously. This should make the reconstructed sampling map $\tilde{\text{F}}$ an ideal tool for use in model-based control.

We also envisage a closed loop identification of SSMs and backbone curves, similar to control-based continuation techniques [29]. In this case, a measure of invariance derived from equation (5.8) would serve as a test functional.

## Supplementary Material

Nonlinear model identification and spectral submanifolds for multi-degree-of-freedom mechanical vibrations

## Supplementary Material

Vibration data of the clamped-clamped beam

## Data accessibility

The vibration data corresponding to the clamped–clamped beam is included as an electronic supplementary material.
