# Supplementary material for: Nonlinear model identification and spectral submanifolds for multi-degree-of-freedom mechanical vibrations
Source: Proc Math Phys Eng Sci. 2017 Jun 14;473(2202):20160759. doi: 10.1098/rspa.2016.0759 (PMC5493940; doi:10.1098/rspa.2016.0759)
Supplement: Nonlinear model identification and spectral submanifolds for multi-degree-of-freedom mechanical vibrations [file rspa20160759supp1.pdf]

Nonlinear model identification and spectral submanifolds for multi-degree-of-freedom mechanical vibrations

Electronic Supplementary Material

Robert Szalai[1,*], *David Ehrhardt*[2] *and George Haller*[3]

[1]Department of Engineering Mathematics, University of Bristol, Merchant Venturers Building, Woodland Road, Bristol BS8 1UB, United Kingdom

[2]Department of Mechanical Engineering, University of Bristol, Queen's Building, University Walk, Clifton BS8 1TR, United Kingdom

[3]Institute for Mechanical Systems, ETH Zürich, Leonhardstrasse 21, Zürich, 8092, Switzerland.

A Proof of Theorem 1 (main text)

By the relationship

$$\boldsymbol{F}\circ\boldsymbol{W}=\boldsymbol{W}\circ\boldsymbol{R}, \tag{1}$$

we need to solve the algebraic equation

$$\boldsymbol{\Lambda}\boldsymbol{W}+\boldsymbol{G}\circ\boldsymbol{W}=\boldsymbol{W}\circ\boldsymbol{R} \tag{2}$$

for the unknown Taylor coefficients $w_j^{\boldsymbol{s}}$ and $r_j^{\boldsymbol{s}}$. We carry this out step by step for increasing powers of $\boldsymbol{z}$:

$\mathcal{O}\left(|\boldsymbol{z}|\right)$: Since the Taylor series of $\boldsymbol{G}$ starts with second-order terms, the first-order monomials of $\boldsymbol{z}$ arising from substitution into (2) satisfy $\boldsymbol{\Lambda}\boldsymbol{W}=\boldsymbol{W}\circ\boldsymbol{R}$, which simplifies to $\boldsymbol{\Lambda}\boldsymbol{W}=\boldsymbol{W}\boldsymbol{\Lambda}$, because the linear part of $\boldsymbol{R}$ is equal to the diag $\{\mu_\ell,\bar{\mu}_\ell\}$ block of the diagonal matrix $\boldsymbol{\Lambda}$. Consequently, (2) can be written at leading order as

$$\mu_j w_j^{(1,0)}=w_j^{(1,0)}\mu_\ell,\qquad \bar{\mu}_j w_{j+1}^{(0,1)}=w_{j+1}^{(0,1)}\bar{\mu}_\ell, \tag{3}$$

whose simplest solution is

$$w_j^{(1,0)}=\delta_{j\ell},\qquad w_j^{(0,1)}=\delta_{j(\ell+1)}, \tag{4}$$

with $\delta_{j\ell}$ denoting the Kronecker delta. This proves the formulas for the first-order coefficients in Theorem 2 (in the main text). We note that $w_\ell^{(1,0)}$ and $w_{\ell+1}^{(0,1)}$ are only determined up to a constant, which we have chosen to be equal to 1.

*Corresponding author. Email: r.szalai@bristol.ac.uk

$\mathcal{O}\left(|\boldsymbol{z}|^2\right)$: Since $r_j^{\boldsymbol{s}}=0$ for $|\boldsymbol{s}|=2$ by (??), the quadratic terms in $|\boldsymbol{z}|$ on the right-hand side of (2) only arise from the substitution of linear terms of $\boldsymbol{R}(\boldsymbol{z})$ into the quadratic terms of $\boldsymbol{W}(\boldsymbol{z})$. As a consequence, equating the coefficients of $\mathcal{O}\left(|\boldsymbol{z}|^{\mathbf{2}}\right)$ terms on both sides of (2) gives the equation

$$\mu_j w_j^{(s_1,s_2)} + g_j^{(s_1@\ell,s_2@(\ell+1))} = \mu_\ell^{s_1}\bar{\mu}_\ell^{s_2} w_j^{(s_1,s_2)}, \qquad |\boldsymbol{s}|=2, \tag{5}$$

whose solution for $w_j^{(s_1,s_2)}$ is

$$w_j^{(s_1,s_2)} = \frac{g_j^{(s_1@\ell,s_2@(\ell+1))}}{\mu_\ell^{s_1}\bar{\mu}_\ell^{s_2}-\mu_j}, \qquad |\boldsymbol{s}|=2, \tag{6}$$

proving the formulas for the second-order coefficients $w_j^{(2,0)}$, $w_j^{(1,1)}$ and $w_j^{(0,2)}$ in the statement of Theorem 2 (main text). Note that the denominator in (6) is guaranteed to be nonzero by the nonresonance condition (equation (17), main text)

$$\mu_\ell^{s_1}\bar{\mu}_\ell^{s_2} \neq \mu_j, \qquad \forall j\neq \ell,\ell+1, \qquad 2\leq s_1+s_2\leq \sigma\left(\mathcal{E}\right). \tag{7}$$

$\mathcal{O}\left(|\boldsymbol{z}|^3\right)$: We write out the jth coordinates in the three terms of eq. (2) in detail to obtain the following cubic terms:

$$\begin{aligned}
(\boldsymbol{\Lambda W})_j^{(3)} &= \mu_j\left(w_j^{(3,0)}z_\ell^3 + w_j^{(2,1)}z_\ell^2\bar{z}_\ell + w_j^{(1,2)}z_\ell\bar{z}_\ell^2 + w_j^{(0,3)}\bar{z}_\ell^3\right), && (8)\\
(\boldsymbol{G}\circ\boldsymbol{W})_j^{(3)} &= \sum_{\substack{|\boldsymbol{m}|=2\\ \boldsymbol{y}^{\boldsymbol{m}}=\mathcal{O}\left(|\boldsymbol{z}|^{\mathbf{3}}\right)}} g_j^{\boldsymbol{m}}\boldsymbol{y}^{\boldsymbol{m}} + \sum_{\substack{|\boldsymbol{m}|=3\\ \boldsymbol{y}^{\boldsymbol{m}}=\mathcal{O}\left(|\boldsymbol{z}|^{\mathbf{3}}\right)}} g_j^{\boldsymbol{m}}\boldsymbol{y}^{\boldsymbol{m}}, && (9)\\
(\boldsymbol{W}\circ\boldsymbol{R})_j^{(3)} &= \sum_{\substack{|\boldsymbol{s}|=1,2,3\\ \boldsymbol{r}^{\boldsymbol{s}}=\mathcal{O}\left(|\boldsymbol{z}|^{\mathbf{3}}\right)}} w_j^{(s_1,s_2)} r_\ell^{s_1}\bar{r}_\ell^{s_2}\\
&= \sum_{\substack{|\boldsymbol{s}|=1,2,3\\ \boldsymbol{r}^{\boldsymbol{s}}=\mathcal{O}\left(|\boldsymbol{z}|^{\mathbf{3}}\right)}} w_j^{(s_1,s_2)}\left(\mu_\ell z_\ell + \beta_\ell z_\ell^2\bar{z}_\ell\right)^{s_1}\left(\bar{\mu}_\ell\bar{z}_\ell + \bar{\beta}_\ell z_\ell\bar{z}_\ell^2\right)^{s_2}\\
&= w_j^{(3,0)}\mu_\ell^3 z_\ell^3 + w_j^{(0,3)}\bar{\mu}_\ell^3\bar{z}_\ell^3 + w_j^{(2,1)}\mu_\ell^2\bar{\mu}_\ell z_\ell^2\bar{z}_\ell\\
&\quad + w_j^{(1,2)}\mu_\ell\bar{\mu}_\ell^2 z_\ell\bar{z}_\ell^2 + w_j^{(1,0)}\beta_\ell z_\ell^2\bar{z}_\ell + w_j^{(0,1)}\bar{\beta}_\ell z_\ell\bar{z}_\ell^2. && (10)
\end{aligned}$$

We now write out the individual terms in eq. (9). For $\boldsymbol{y}=\boldsymbol{W}(\boldsymbol{z})$, we have

$$\begin{aligned}
y_p y_q &= \left(\delta_{p\ell}z_\ell + \delta_{p(\ell+1)}\bar{z}_\ell + w_p^{(2,0)}z_\ell^2 + w_p^{(1,1)}z_\ell\bar{z}_\ell + w_p^{(0,2)}\bar{z}_\ell^2\right)\\
&\quad\times\left(\delta_{q\ell}z_\ell + \delta_{q(\ell+1)}\bar{z}_\ell + w_q^{(2,0)}z_\ell^2 + w_q^{(1,1)}z_\ell\bar{z}_\ell + w_q^{(0,2)}\bar{z}_\ell^2\right)\\
&= \left(\delta_{p\ell}w_q^{(2,0)} + \delta_{q\ell}w_p^{(2,0)}\right)z_\ell^3 + \left(\delta_{p\ell}w_q^{(1,1)} + \delta_{q\ell}w_p^{(1,1)} + \delta_{p(\ell+1)}w_q^{(2,0)} + \delta_{q(\ell+1)}w_p^{(2,0)}\right)z_\ell^2\bar{z}_\ell\\
&\quad + \left(\delta_{p\ell}w_q^{(0,2)} + \delta_{q\ell}w_p^{(0,2)} + \delta_{p(\ell+1)}w_q^{(1,1)} + \delta_{q(\ell+1)}w_p^{(1,1)}\right)z_\ell\bar{z}_\ell^2 + \left(\delta_{p(\ell+1)}w_q^{(0,2)} + \delta_{q(\ell+1)}w_p^{(0,2)}\right)\bar{z}_\ell^3\\
&\quad + \mathcal{O}\left(|\boldsymbol{z}|^2, |\boldsymbol{z}|^4\right),
\end{aligned}$$

thus, for the first sum in (9), we obtain

$$\begin{aligned}
\sum_{\substack{|\boldsymbol{m}|=2\\ y^{\boldsymbol{m}}=\mathcal{O}\left(|\boldsymbol{z}|^3\right)}} g_j^{\boldsymbol{m}}\boldsymbol{y}^{\boldsymbol{m}} \;=\; & \sum_{p,q=1}^{2\nu} \frac{g_j^{(1@p,1@q)}}{2-\delta_{pq}} \left[\left(\delta_{p\ell}w_q^{(2,0)}+\delta_{q\ell}w_p^{(2,0)}\right)z_\ell^3+\left(\delta_{p(\ell+1)}w_q^{(0,2)}+\delta_{q(\ell+1)}w_p^{(0,2)}\right)\bar{z}_\ell^3\right]\\
&+\sum_{p,q=1}^{2\nu} \frac{g_j^{(1@p,1@q)}}{2-\delta_{pq}} \left[\delta_{p\ell}w_q^{(1,1)}+\delta_{q\ell}w_p^{(1,1)}+\delta_{p(\ell+1)}w_q^{(2,0)}+\delta_{q(\ell+1)}w_p^{(2,0)}\right]z_\ell^2\bar{z}_\ell\\
&+\sum_{p,q=1}^{2\nu} \frac{g_j^{(1@p,1@q)}}{2-\delta_{pq}} \left[\delta_{p\ell}w_q^{(0,2)}+\delta_{q\ell}w_p^{(0,2)}+\delta_{p(\ell+1)}w_q^{(1,1)}+\delta_{q(\ell+1)}w_p^{(1,1)}\right]z_\ell\bar{z}_\ell^2\\
=2\; & \sum_{q=1}^{2\nu}\frac{g_j^{(1@\ell,1@q)}}{2-\delta_{\ell q}}w_q^{(2,0)}z_\ell^3+2\sum_{q=1}^{2\nu}\frac{g_j^{(1@(\ell+1),1@q)}}{2-\delta_{(\ell+1)q}}w_q^{(0,2)}\bar{z}_\ell^3\\
&+2\sum_{q=1}^{2\nu}\left[\frac{g_j^{(1@\ell,1@q)}}{2-\delta_{\ell q}}w_q^{(1,1)}+\frac{g_j^{(1@(\ell+1),1@q)}}{2-\delta_{(\ell+1)q}}w_q^{(2,0)}\right]z_\ell^2\bar{z}_\ell\\
&+2\sum_{q=1}^{2\nu}\left[\frac{g_j^{(1@\ell,1@q)}}{2-\delta_{\ell q}}w_q^{(0,2)}+\frac{g_j^{(1@(\ell+1),1@q)}}{2-\delta_{(\ell+1)q}}w_q^{(1,1)}\right]z_\ell\bar{z}_\ell^2. \qquad (11)
\end{aligned}$$

For the second sum in (9), we have

$$\begin{aligned}
\sum_{\substack{|\boldsymbol{m}|=3\\ y^{\boldsymbol{m}}=\mathcal{O}\left(|\boldsymbol{z}|^3\right)}} g_j^{\boldsymbol{m}}\boldsymbol{y}^{\boldsymbol{m}} \;=\; & \underbrace{\sum_{\substack{p=1\\ y^{\boldsymbol{m}}=\mathcal{O}\left(|\boldsymbol{z}|^3\right)}}^{2\nu} g_j^{(3@p)}y_p^3}_{A} + \underbrace{\sum_{\substack{p,q=1\\ p\neq q\\ y^{\boldsymbol{m}}=\mathcal{O}\left(|\boldsymbol{z}|^3\right)}}^{2\nu} g_j^{(2@p,1@q)}y_p^2y_q}_{B} \qquad (12)\\
&+\underbrace{\sum_{\substack{p,q,u=1\\ p\neq q;\, p,q\neq u\\ y^{\boldsymbol{m}}=\mathcal{O}\left(|\boldsymbol{z}|^3\right)}}^{2\nu} g_j^{(1@p,1@q,1@u)}y_py_qy_u}_{C}\,.
\end{aligned}$$

Working out these expressions in detail, we find that

$$
\begin{aligned}
A &= \sum_{p=1}^{2\nu} g_j^{(3@p)} \left(\delta_{p\ell}^3 z_\ell^3 + 3\delta_{p\ell}^2 \delta_{p(\ell+1)} z_\ell^2 \bar{z}_\ell + 3\delta_{p\ell}\delta_{p(\ell+1)}^2 z_\ell \bar{z}_\ell^2 + \delta_{p(\ell+1)}^3 \bar{z}_\ell^3\right) \\
&= g_j^{(3@\ell)} z_\ell^3 + g_j^{(3@(\ell+1))} \bar{z}_\ell^3, \\
B &= \sum_{\substack{p,q=1 \\ p\neq q}}^{2\nu} g_j^{(2@p,1@q)} \left(\delta_{p\ell}^2 z_\ell^2 + 2\delta_{p\ell}\delta_{p(\ell+1)} z_\ell \bar{z}_\ell + \delta_{p(\ell+1)}^2 \bar{z}_\ell^2\right)\left(\delta_{q\ell} z_\ell + \delta_{q(\ell+1)} \bar{z}_\ell\right) \\
&= \sum_{\substack{p,q=1 \\ p\neq q}}^{2\nu} g_j^{(2@p,1@q)} \left(\delta_{p\ell}^2 \delta_{q\ell} z_\ell^3 + 2\delta_{p\ell}\delta_{q\ell}\delta_{p(\ell+1)} z_\ell^2 \bar{z}_\ell + \delta_{q\ell}\delta_{p(\ell+1)}^2 z_\ell \bar{z}_\ell^2\right) \\
&+ \sum_{\substack{p,q=1 \\ p\neq q}}^{2\nu} g_j^{(2@p,1@q)} \left(\delta_{p\ell}^2 \delta_{q(\ell+1)} z_\ell^2 \bar{z}_\ell + 2\delta_{p\ell}\delta_{p(\ell+1)}\delta_{q(\ell+1)} z_\ell \bar{z}_\ell^2 + \delta_{p(\ell+1)}^2 \delta_{q(\ell+1)} \bar{z}_\ell^3\right) \\
&= g_j^{(2@\ell,1@(\ell+1))} z_\ell^2 \bar{z}_\ell + g_j^{(2@(\ell+1),1@\ell)} z_\ell \bar{z}_\ell^2, \\
C &= \sum_{\substack{p,q,u=1 \\ p\neq q;\, p,q\neq u}}^{2\nu} g_j^{(1@p,1@q,1@u)} \left(\delta_{p\ell} z_\ell + \delta_{p(\ell+1)} \bar{z}_\ell\right)\left(\delta_{q\ell} z_\ell + \delta_{q(\ell+1)} \bar{z}_\ell\right)\left(\delta_{u\ell} z_\ell + \delta_{u(\ell+1)} \bar{z}_\ell\right) \\
&= 0.
\end{aligned} \tag{13}
$$

Substituting the expressions for A, B and C into (12), then substituting (12) and (11) into the invariance condition (2), we equate equal powers of $\boldsymbol{z}$ to obtain the following linear equations for the cubic coefficients of the mapping $\boldsymbol{W}$ and of the mapping $\boldsymbol{R}$:

$$
\begin{aligned}
\mathcal{O}\left(z_\ell^3\right) &: \mu_j w_j^{(3,0)} + 2\sum_{q=1}^{2\nu} \frac{g_j^{(1@\ell,1@q)}}{2-\delta_{\ell q}} w_q^{(2,0)} + g_j^{(3@\ell)} \\
&\quad = w_j^{(3,0)} \mu_\ell^3, \\
\mathcal{O}\left(z_\ell^2 \bar{z}_\ell\right) &: \mu_j w_j^{(2,1)} + 2\sum_{q=1}^{2\nu} \left[\frac{g_j^{(1@\ell,1@q)}}{2-\delta_{\ell q}} w_q^{(1,1)} + \frac{g_j^{(1@(\ell+1),1@q)}}{2-\delta_{(\ell+1)q}} w_q^{(2,0)}\right] + g_j^{(2@\ell,1@(\ell+1))} \\
&\quad = w_j^{(2,1)} \mu_\ell^2 \bar{\mu}_\ell + w_j^{(1,0)} \beta_\ell, \\
\mathcal{O}\left(z_\ell \bar{z}_\ell^2\right) &: \mu_j w_j^{(1,2)} + 2\sum_{q=1}^{2\nu} \frac{g_j^{(1@(\ell+1),1@q)}}{2-\delta_{(\ell+1)q}} + g_j^{(2@(\ell+1),1@\ell)} \\
&\quad = w_j^{(1,2)} \mu_\ell \bar{\mu}_\ell^2 + w_j^{(0,1)} \bar{\beta}_\ell, \\
\mathcal{O}\left(\bar{z}_\ell^3\right) &: \mu_j w_j^{(0,3)} + 2\sum_{q=1}^{2\nu} \frac{g_j^{(1@(\ell+1),1@q)}}{2-\delta_{(\ell+1)q}} w_q^{(0,2)} + g_j^{(3@(\ell+1))} \\
&\quad = w_j^{(0,3)} \bar{\mu}_\ell^3.
\end{aligned} \tag{14}
$$

From the first and last equation in (14), we obtain

$$\begin{aligned} w_j^{(3,0)} &= \frac{\sum_{q=1}^{2\nu}\left(1+\delta_{\ell q}\right)g_j^{(1@\ell,1@q)}w_q^{(2,0)}+g_j^{(3@\ell)}}{\mu_\ell^3-\mu_j}, \\ w_j^{(0,3)} &= \frac{\sum_{q=1}^{2\nu}\left(1+\delta_{(\ell+1)q}\right)g_j^{(1@(\ell+1),1@q)}w_q^{(0,2)}+g_j^{(3@(\ell+1))}}{\bar{\mu}_\ell^3-\mu_j}. \end{aligned} \tag{15}$$

We select $j\neq\ell$ and assume that there is no first-order near-resonance (or exact resonance) involving the eigenvalues μ_ℓ and μ_j (stated as $\mu_j\not\approx\mu_\ell$ under the assumptions of the theorem). Recalling $w_j^{(1,0)}=\delta_{j\ell}$, we then obtain from the second equation of (14) that

$$w_j^{(2,1)}=\frac{\sum_{q=1}^{2\nu}\left[\left(1+\delta_{\ell q}\right)g_j^{(1@\ell,1@q)}w_q^{(1,1)}+\left(1+\delta_{(\ell+1)q}\right)g_j^{(1@(\ell+1),1@q)}w_q^{(2,0)}\right]+g_j^{(2@\ell,1@(\ell+1))}}{\mu_\ell^2\bar{\mu}_\ell-\mu_j}, \tag{16}$$

whenever $\delta_{j\ell}=0$. Similarly, selecting $j\neq\ell+1$, assuming no first-order near-resonance (or exact resonance) involving the eigenvalues $\mu_{\ell+1}$ and μ_j (i.e., $\mu_j\not\approx\mu_{\ell+1}$), and recalling $w_j^{(0,1)}=\delta_{j(\ell+1)}$, we obtain from the third equation of (14) that

$$w_j^{(1,2)} = \frac{\sum_{q=1}^{2\nu}\left[\left(1+\delta_{\ell q}\right)g_j^{(1@\ell,1@q)}w_q^{(0,2)}+\left(1+\delta_{(\ell+1)q}\right)g_j^{(1@(\ell+1),1@q)}w_q^{(1,1)}\right]+g_j^{(2@(\ell+1),1@\ell)}}{\mu_\ell\bar{\mu}_\ell^2-\mu_j}, \tag{17}$$

whenever $\delta_{j(\ell+1)}=0$. Next we select $j=\ell$ in the second equation of (14), and select $j=\ell+1$ in the third equation of (14). These choices force us to select

$$w_\ell^{(2,1)}=0,\quad \delta_{j\ell}=1,\qquad w_{\ell+1}^{(1,2)}=0,\quad \delta_{j(\ell+1)}=1, \tag{18}$$

in these equations to avoid small denominators arising from the near-resonances. Then the second equation of (14) with $j=\ell$ gives the solution

$$\beta_\ell=\sum_{q=1}^{2\nu}\left[\left(1+\delta_{\ell q}\right)g_\ell^{(1@\ell,1@q)}w_q^{(1,1)}+\left(1+\delta_{(\ell+1)q}\right)g_\ell^{(1@(\ell+1),1@q)}w_q^{(2,0)}\right]+g_\ell^{(2@\ell,1@(\ell+1))}. \tag{19}$$

But equations (15)-(19) prove the formulas for the cubic coefficients of W_j and β_ℓ in the statement of Theorem 2 (main text).

B Analogous results for continuous dynamical systems

Here we discuss spectral submanifolds, backbone curves and their leading-order computation for continuous dynamical systems. The formulas we derive are useful for benchmarking our data-based SSM and backbone-curve approach on exactly known mechanical models. The concepts and formulas derived here, however, are also of independent interest in computing the dynamics on SSMs in analytically defined mechanical models.

We start with the continuous analogue of the the complex mapping

$$\boldsymbol{y}_{k+1}=\boldsymbol{\Lambda}\boldsymbol{y}_k+\boldsymbol{G}(\boldsymbol{y}_k),\qquad \boldsymbol{\Lambda}=\mathrm{diag}(\mu_1,\mu_2\dots,\mu_{2\nu})=\boldsymbol{V}^{-1}\boldsymbol{A}\boldsymbol{V},\qquad \mu_{2l}=\bar{\mu}_{2l-1},\quad l=1,\dots,\nu, \tag{20}$$

which is a complex differential equation of the form

$$\dot{\boldsymbol{y}} = \boldsymbol{\Lambda}\boldsymbol{y} + \boldsymbol{G}(\boldsymbol{y}), \qquad \boldsymbol{y} \in \mathbb{C}^{2\nu}, \qquad \boldsymbol{\Lambda} = \mathrm{diag}(\lambda_1, \ldots, \lambda_{2\nu}), \qquad \lambda_{2l-1} = \bar{\lambda}_{2l}, \quad l = 1, \ldots, \nu, \tag{21}$$
$$\boldsymbol{G}(\boldsymbol{y}) = \mathcal{O}\left(|\boldsymbol{y}|^2\right),$$

with a fixed point at $\boldsymbol{y} = \boldsymbol{0}$, and with a class C^r function $\boldsymbol{G}$. The eigenvalues of $\boldsymbol{\Lambda}$ are ordered so that

$$\mathrm{Re}\lambda_{2\nu} \leq \ldots \leq \mathrm{Re}\lambda_1 < 0, \tag{22}$$

and hence $\boldsymbol{y} = \boldsymbol{0}$ is asymptotically stable. If (21) is the equivalent first-order complexified form of a mechanical system of the form

$$\boldsymbol{M}(\boldsymbol{q})\ddot{\boldsymbol{q}} - \boldsymbol{f}(\boldsymbol{q}, \dot{\boldsymbol{q}}) = \boldsymbol{0}, \qquad \boldsymbol{f}(\boldsymbol{0}, \boldsymbol{0}) = \boldsymbol{0}, \tag{23}$$

then we specifically have $\nu = n$. If, furthermore, the mechanical system has linear and weak proportional damping, then we can write

$$\mathrm{Re}\lambda_l = -\zeta_l\omega_l, \qquad \mathrm{Im}\lambda_l = \sqrt{1 - \zeta_l^2}\omega_l, \tag{24}$$

with ζ_l and ω_l denoting Lehr's damping ratio and undamped natural frequency, respectively, for the lth mode of the linearised system at the $\boldsymbol{q} = \boldsymbol{0}$ equilibrium.

Finally, we assume that $\mathcal{E}$ is a two-dimensional spectral subspace (eigenspace) of the operator $\boldsymbol{\Lambda}$, corresponding to the complex pair of simple eigenvalues $\lambda_\ell = \overline{\lambda}_{\ell+1}$ for some $\ell \in [1, 2\nu - 1]$.

B.1 Existence and uniqueness of SSMs

Following [2], we address this issue via the following definition:

Definition 1. A *spectral submanifold* (SSM) $W(\mathcal{E})$ corresponding to a spectral subspace $\mathcal{E}$ of $\boldsymbol{\Lambda}$ is

(i) an invariant manifold of the dynamical system (21) that is tangent to $\mathcal{E}$ at $\boldsymbol{y} = \boldsymbol{0}$ and has the same dimension as $\mathcal{E}$;

(ii) strictly smoother than any other invariant manifold of (21) satisfying (i).

We now recall from Haller and Ponsioen [2] the specific existence and uniqueness result pertaining to two-dimensional SSMs, deducible from the more general results of Cabré et al. [1]. The *relative spectral quotient* of $\mathcal{E}$ is now defined as the positive integer

$$\sigma(\mathcal{E}) = \mathrm{Int}\left[\frac{\min\limits_{l \neq \ell, \ell+1} \mathrm{Re}\lambda_l}{\mathrm{Re}\lambda_\ell}\right] \in \mathbb{N}^+, \tag{25}$$

whose meaning is the same as pointed out after formula (25) for mappings. In case of a proportionally damped mechanical system, one may use the formulas (24) and conclude that Remark 1 continues to provide the correct specific form of $\sigma(\mathcal{E})$ in this case.

We again assume that

$$\sigma(\mathcal{E}) \leq r, \tag{26}$$

and that no resonance relationships up to order $\sigma(\mathcal{E})$ hold between the eigenvalues $\lambda_\ell, \overline{\lambda}_{\ell+1}$ and the rest of the spectrum of $\boldsymbol{\Lambda}$, i.e.,

$$s_1\lambda_\ell + s_2\bar{\lambda}_\ell \neq \lambda_j, \qquad \forall j \neq \ell, \ell+1, \qquad 2 \leq s_1 + s_2 \leq \sigma\left(\mathcal{E}\right). \tag{27}$$

The alternative form of this nonresonance condition given in Remark 1 again applies whenever formulas (25) hold.

Theorem 1. *Assume that conditions (26)-(27) are satisfied. Then following statements hold:*

(i) There exists an SSM, $W\left(\mathcal{E}\right)$, for the dynamical system (21) that is tangent to the invariant subspace $\mathcal{E}$ at the $\boldsymbol{y} = \boldsymbol{0}$ fixed point.

(ii) The invariant manifold $W\left(\mathcal{E}\right)$ is class C^r smooth and unique among all two-dimensional, class $C^{\sigma(\mathcal{E})+1}$ invariant manifolds of (21) that are tangent to $\mathcal{E}$ at $\boldsymbol{y} = \boldsymbol{0}$.

(iii) The SSM $W\left(\mathcal{E}\right)$ can be viewed as a C^r immersion of an open set $\mathcal{U} \subset \mathbb{C}^2$ into the phase space $\mathbb{C}^{2\nu}$ of system (21) via a map

$$\boldsymbol{W} : \mathcal{U} \subset \mathbb{C}^2 \;\; \to \;\; \mathbb{C}^{2\nu}, \qquad \boldsymbol{W}\left(\mathcal{U}\right) = W\left(\mathcal{E}\right). \tag{28}$$

(iv) There exists C^r polynomial function $\boldsymbol{R}\colon \mathcal{U} \to \mathcal{U}$ such that

$$\boldsymbol{\Lambda W} + \boldsymbol{G} \circ \boldsymbol{W} = D\boldsymbol{W}\boldsymbol{R}, \tag{29}$$

i.e., the dynamics on the SSM, expressed in coordinates $\boldsymbol{z} = (z_\ell, \bar{z}_\ell) \in \mathcal{U}$, is governed by the polynomial ODE

$$\dot{\boldsymbol{z}} = \boldsymbol{R}(\boldsymbol{z}), \qquad D\boldsymbol{R}(0) = \mathrm{diag}(\lambda_\ell, \bar{\lambda}_\ell),$$

whose right-hand side has only terms up to order $\mathcal{O}\left(|\boldsymbol{z}|^{\sigma(\mathcal{E})}\right)$.

(v) Under the further internal non-resonance assumption

$$s_1\lambda_\ell + s_2\bar{\lambda}_\ell \neq \lambda_j, \qquad j = \ell, \ell+1, \qquad 2 \leq s_1 + s_2 \leq \sigma\left(\mathcal{E}_\ell\right), \tag{30}$$

within $\mathcal{E}$, the mapping $\boldsymbol{W}$ in 28 can be selected such that the jth coordinate component R_j of $\boldsymbol{R}$ does not contain the term $(z_\ell^{s_1}, \bar{z}_\ell^{s_2})$.

Proof. This is merely the re-statement of the main theorem of Haller and Ponsioen [2] (deduced from Cabré et al. [1]) for the case of a two-dimensional SSM corresponding to a simple pair of complex eigenvalues with negative real parts. □

B.2 Backbone curves and their computation

When the spectral subspace $\mathcal{E}$ of (21) is lightly damped ($|\mathrm{Re}\lambda_\ell| \ll 1$), the low-order near-resonance relationships

$$2\lambda_\ell + \bar{\lambda}_\ell \approx \lambda_\ell, \quad \lambda_\ell + 2\bar{\lambda}_\ell \approx \bar{\lambda}_\ell$$

always hold. As in the case of mappings, this prompts us to seek the polynomial dynamics on the SSM (cf. statement (iv) of Theorem 1) in the form

$$\dot{\boldsymbol{z}} = \boldsymbol{R}(\boldsymbol{z}) = \begin{pmatrix} \lambda_\ell z_\ell + \beta_\ell z_\ell^2 \bar{z}_\ell \\ \bar{\lambda}_\ell \bar{z}_\ell + \bar{\beta}_\ell z_\ell \bar{z}_\ell^{\,2} \end{pmatrix}. \tag{31}$$

Introducing polar coordinates $z = re^{i\theta}$, we can further transform (31) to the real amplitude-phase equations

$$\dot{\rho} = \rho\left(\mathrm{Re}\lambda_\ell + \mathrm{Re}\beta_\ell\rho^2\right), \tag{32}$$

$$\dot{\theta} = \mathrm{Im}\lambda_\ell + \mathrm{Im}\beta_\ell\rho^2. \tag{33}$$

Equation (33) gives instantaneous frequency of nonlinear oscillations as

$$\omega(\rho) = \mathrm{Im}\lambda_\ell + \mathrm{Im}\beta_\ell\rho^2, \tag{34}$$

whereas as instantaneous amplitude $\mathrm{Amp}(\rho)$ of the vibration can be calculated as

$$\mathrm{Amp}(\rho) = \frac{1}{2\pi}\int_0^{2\pi} |\boldsymbol{V}\,\boldsymbol{W}(\boldsymbol{z}(\rho,\theta))|\, d\theta, \tag{35}$$

where $\boldsymbol{W}$ is the mapping featured in statement (iii) of Theorem 1, and $\boldsymbol{V}$ is the linear mapping that transform the original, first-order dynamical system to its standard complex form (21). With the quantities defined in (34) and (35), the definition of a backbone curve $\mathcal{B}_\ell$ given in Definition 2 carries over without change to our present context. Again, to compute the backbone curve

$$\mathcal{B}_\ell = \{\omega(\rho_\ell), \mathrm{Amp}(\rho_\ell)\}_{\rho_\ell\in\mathbb{R}^+} \subset \mathbb{R}^2, \tag{36}$$

we need to find expressions for the complex coefficient β_ℓ and the mapping $\boldsymbol{W}(\boldsymbol{z})$, as the eigenvalue λ_ℓ is assumed to be known.

To this end, we seek the Taylor series coefficients of the jth coordinate functions, $W_j(\boldsymbol{z}) \in \mathbb{C}$, $j = 1,\ldots,2\nu$, of the mapping $\boldsymbol{W}(\boldsymbol{z})$, and the third-order Taylor coefficient $\beta_\ell \in \mathbb{C}$ of the polynomial function $\boldsymbol{R}(\boldsymbol{z})$ defined in (31). These unknowns will again be expressed as functions of the jth coordinate functions $G_j(\boldsymbol{y}) \in \mathbb{C}$, $j = 1,\ldots,2\nu$, of the nonlinear part $\boldsymbol{G}(\boldsymbol{y})$ of the right-hand side of the dynamical system (21). Using the same notation as in Theorem (2), we obtain the following expressions for the required Taylor coefficients.

Theorem 2. *Suppose that the assumptions of Theorem 1 hold but with the strengthened version*

$$s_1\lambda_\ell + s_2\bar{\lambda}_\ell \not\approx \lambda_j, \qquad \forall j \neq \ell, \ell+1, \qquad 1 \leq s_1 + s_2 \leq \sigma\left(\mathcal{E}\right) \tag{37}$$

of the external non-resonance condition (27). Then, for any $j \in [1, 2\nu]$, the jth coordinate function W_j of the mapping $\boldsymbol{W}$ in (28) and the cubic Taylor coefficient β_ℓ of the conjugate map $\boldsymbol{R}$ in (29) are given by the following formulas:

$$w_j^{(1,0)} = \delta_{j\ell}, \qquad w_j^{(0,1)} = \delta_{j(\ell+1)},$$

$$w_j^{(2,0)} = \frac{g_j^{(2@\ell)}}{2\lambda_l - \lambda_j}, \qquad w_j^{(1,1)} = \frac{g_j^{(1@\ell,1@(\ell+1))}}{\lambda_\ell + \bar{\lambda}_\ell - \lambda_j}, \qquad w_j^{(0,2)} = \frac{g_j^{(2@(\ell+1))}}{2\bar{\lambda}_l - \lambda_j},$$

$$w_j^{(3,0)} = \frac{\sum_{q=1}^{2\nu}\left(1+\delta_{\ell q}\right) g_j^{(1@\ell,1@q)} w_q^{(2,0)} + g_j^{(3@\ell)}}{3\lambda_l - \lambda_j}, \qquad w_j^{(0,3)} = \frac{\sum_{q=1}^{2\nu}\left(1+\delta_{(\ell+1)q}\right) g_j^{(1@(\ell+1),1@q)} w_q^{(0,2)} + g_j^{(3@(\ell+1))}}{3\bar{\lambda}_l - \lambda_j}.$$

$$w_j^{(2,1)} = \left(1-\delta_{j\ell}\right)\frac{\sum_{q=1}^{2\nu}\left[\left(1+\delta_{\ell q}\right) g_j^{(1@\ell,1@q)} w_q^{(1,1)} + \left(1+\delta_{(\ell+1)q}\right) g_j^{(1@(\ell+1),1@q)} w_q^{(2,0)}\right] + g_j^{(2@\ell,1@(\ell+1))}}{2\lambda_\ell + \bar{\lambda}_\ell - \lambda_j},$$

$$w_j^{(1,2)} = \left(1-\delta_{j(\ell+1)}\right) \frac{\sum_{q=1}^{2\nu}\left[\left(1+\delta_{\ell q}\right) g_j^{(1@\ell,1@q)} w_q^{(0,2)} + \left(1+\delta_{(\ell+1)q}\right) g_j^{(1@(\ell+1),1@q)} w_q^{(1,1)}\right] + g_j^{(2@(\ell+1),1@\ell)}}{\lambda_\ell + 2\bar{\lambda}_\ell - \lambda_j},$$

$$\beta_\ell = \sum_{q=1}^{2\nu}\left[\left(1+\delta_{\ell q}\right) g_\ell^{(1@\ell,1@q)} w_q^{(1,1)} + \left(1+\delta_{(\ell+1)q}\right) g_\ell^{(1@(\ell+1),1@q)} w_q^{(2,0)}\right] + g_\ell^{(2@\ell,1@(\ell+1))}.$$

Proof. The algebraic equation (29) is similar to the equation (1), which we have solved in detail up to cubic order in the proof of Theorem 2 (main text). The first difference between the two equations is that the term $\boldsymbol{\Lambda W}$ in (1) has the jth component

$$\left(\boldsymbol{\Lambda W}\right)_j = \lambda_j \sum_{|\boldsymbol{s}|\geq 1} w_j^{\boldsymbol{s}} \boldsymbol{z}^{\boldsymbol{s}}, \ \boldsymbol{s}\in\mathbb{N}^2, \qquad w_j^{\boldsymbol{s}}\in\mathbb{C}. \tag{38}$$

The second difference is that instead of $(\boldsymbol{W}\circ\boldsymbol{R})_j$, the jth coordinate component of the right-hand side of (1) is given by

$$\begin{aligned}(D\boldsymbol{W}\circ\boldsymbol{R})_j &= \partial_{z_\ell} W_j(\boldsymbol{z})\left(\lambda_\ell z_\ell + \beta_\ell z_\ell^2 \bar{z}_\ell\right) + \partial_{\bar{z}_\ell} W_j(\boldsymbol{z})\left(\bar{\lambda}_\ell \bar{z}_\ell + \bar{\beta}_\ell z_\ell \bar{z}_\ell^2\right) \\ &= \lambda_\ell w_j^{(1,0)} z_\ell + 2\lambda_\ell w_j^{(2,0)} z_\ell^2 + \lambda_\ell w_j^{(1,1)} z_\ell \bar{z}_\ell + 3\lambda_\ell w_j^{(3,0)} z_\ell^3 \\ &\quad + 2\lambda_\ell w_j^{(2,1)} z_\ell^2 \bar{z}_\ell + \lambda_\ell w_j^{(1,2)} z_\ell \bar{z}_\ell^2 + \beta_\ell w_j^{(1,0)} z_\ell^2 \bar{z}_\ell + \mathcal{O}\left(|z|^4\right) \\ &\quad + \bar{\lambda}_\ell w_j^{(0,1)} \bar{z}_\ell + 2\bar{\lambda}_\ell w_j^{(0,2)} \bar{z}_\ell^2 + \bar{\lambda}_\ell w_j^{(1,1)} z_\ell \bar{z}_\ell + 3\bar{\lambda}_\ell w_j^{(0,3)} \bar{z}_\ell^3 \\ &\quad + 2\bar{\lambda}_\ell w_j^{(1,2)} z_\ell \bar{z}_\ell^2 + \bar{\lambda}_\ell w_j^{(2,1)} z_\ell^2 \bar{z}_\ell + \bar{\beta}_\ell w_j^{(0,1)} z_\ell \bar{z}_\ell^2 + \mathcal{O}\left(|z|^4\right).\end{aligned} \tag{39}$$

Substituting formulas (38)-(39) into (1), and using the expression for $(\boldsymbol{W}\circ\boldsymbol{G})_j$ from the proof of Theorem 2 (main text), we obtain the formulas in the statement of Theorem (2) after comparing equal powers of $\boldsymbol{z}$ up to cubic order. □

C Analytic SSM and backbone calculations for Example 1

To compute the SSMs $W(E_1)$and $W(E_3)$ in Example 1, we transform equation

$$\begin{aligned}\dot{x}_1 &= v_1, \\ \dot{x}_2 &= v_2, \\ \dot{v}_1 &= -cv_1 - k_0 x_1 - \kappa x_1^3 - k_0(x_1 - x_2) - c(v_1 - v_2), \\ \dot{v}_2 &= -cv_2 - k_0 x_2 - k_0(x_2 - x_1) - c(v_2 - v_1)\end{aligned} \tag{40}$$

to its complex standard form (21). This involves the coordinate change $\boldsymbol{x} = (x_1, x_2, v_1, v_2)^T = \boldsymbol{V y}$, where the matrix $\boldsymbol{V}$ of eigenvectors and its inverse are

$$\boldsymbol{V} = \begin{pmatrix} 1 & 1 & 1 & 1 \\ 1 & 1 & -1 & -1 \\ \lambda_1 & \bar{\lambda}_1 & \lambda_3 & \bar{\lambda}_3 \\ \lambda_1 & \bar{\lambda}_1 & -\lambda_3 & -\bar{\lambda}_3 \end{pmatrix}, \qquad \boldsymbol{V}^{-1} = \begin{pmatrix} -\frac{\bar{\lambda}_1}{2\left(\lambda_1-\bar{\lambda}_1\right)} & -\frac{\bar{\lambda}_1}{2\left(\lambda_1-\bar{\lambda}_1\right)} & \frac{1}{2\left(\lambda_1-\bar{\lambda}_1\right)} & \frac{1}{2\left(\lambda_1-\bar{\lambda}_1\right)} \\ \frac{\lambda_1}{2\left(\lambda_1-\bar{\lambda}_1\right)} & \frac{\lambda_1}{2\left(\lambda_1-\bar{\lambda}_1\right)} & -\frac{1}{2\left(\lambda_1-\bar{\lambda}_1\right)} & -\frac{1}{2\left(\lambda_1-\bar{\lambda}_1\right)} \\ -\frac{\bar{\lambda}_3}{2\left(\lambda_3-\bar{\lambda}_3\right)} & \frac{\bar{\lambda}_3}{2\left(\lambda_3-\bar{\lambda}_3\right)} & \frac{1}{2\left(\lambda_3-\bar{\lambda}_3\right)} & -\frac{1}{2\left(\lambda_3-\bar{\lambda}_3\right)} \\ \frac{\lambda_3}{2\left(\lambda_3-\bar{\lambda}_3\right)} & -\frac{\lambda_3}{2\left(\lambda_3-\bar{\lambda}_3\right)} & -\frac{1}{2\left(\lambda_3-\bar{\lambda}_3\right)} & \frac{1}{2\left(\lambda_3-\bar{\lambda}_3\right)} \end{pmatrix}.$$

The transformed system (40) then takes the form

$$\begin{aligned}
\dot{\boldsymbol{y}} &= \boldsymbol{\Lambda}\boldsymbol{y}+\boldsymbol{G}(\boldsymbol{y}), \qquad \boldsymbol{y}\in\mathbb{C}^4, \qquad \boldsymbol{\Lambda}=\mathrm{diag}(\lambda_1,\lambda_2,\lambda_3,\lambda_4), \quad \boldsymbol{G}(\boldsymbol{y})=\mathcal{O}\left(|\boldsymbol{y}|^2\right),\\
\boldsymbol{G}(\boldsymbol{y}) &= \boldsymbol{V}^{-1}\boldsymbol{f}(\boldsymbol{V}\boldsymbol{y})=-\frac{\kappa}{2}\gamma(\boldsymbol{y})\begin{pmatrix}\frac{1}{\lambda_1-\bar{\lambda}_1}\\ \frac{1}{\bar{\lambda}_1-\lambda_1}\\ \frac{1}{\lambda_3-\bar{\lambda}_3}\\ \frac{1}{\bar{\lambda}_3-\lambda_3}\end{pmatrix}=\frac{i\kappa\,\gamma(\boldsymbol{y})}{4}\begin{pmatrix}\frac{1}{\mathrm{Im}\,\lambda_1}\\ -\frac{1}{\mathrm{Im}\,\lambda_1}\\ \frac{1}{\mathrm{Im}\,\lambda_3}\\ -\frac{1}{\mathrm{Im}\,\lambda_3}\end{pmatrix},
\end{aligned}$$

where

$$\begin{aligned}
\gamma(\boldsymbol{y}) &= y_1^3+y_2^3+y_3^3+y_4^3\\
&\quad +3(y_1^2y_2+y_1^2y_3+y_1^2y_4+y_2^2y_1+y_2^2y_3+y_2^2y_4+y_3^2y_1+y_3^2y_2+y_3^2y_4+y_4^2y_1+y_4^2y_2+y_4^2y_3)\\
&\quad +6(y_1y_2y_3+y_1y_2y_4+y_1y_3y_4+y_2y_3y_4)
\end{aligned}$$

Theorem 2 (this text) then gives the following coefficients for $\ell=1$:

$$w_1^{(1,0)}=1, \quad w_2^{(1,0)}=w_3^{(1,0)}=w_4^{(1,0)}=0, \quad w_2^{(0,1)}=1, \quad w_1^{(0,1)}=w_3^{(0,1)}=w_4^{(0,1)}=0,$$

$$w_j^{(2,0)}=w_j^{(1,1)}=w_j^{(0,2)}=0, \qquad j=1,2,3,4,$$

$$\begin{aligned}
w_1^{(3,0)} &= \frac{i\kappa}{8\lambda_1\mathrm{Im}\,\lambda_1}, & w_2^{(3,0)} &= -\frac{i\kappa}{4\left(3\lambda_1-\bar{\lambda}_1\right)\mathrm{Im}\,\lambda_1},\\
w_3^{(3,0)} &= \frac{i\kappa}{4\left(3\lambda_1-\lambda_3\right)\mathrm{Im}\,\lambda_3}, & w_4^{(3,0)} &= -\frac{i\kappa}{4\left(3\lambda_1-\bar{\lambda}_3\right)\mathrm{Im}\,\lambda_3},\\
w_1^{(0,3)} &= \frac{i\kappa}{4\left(3\bar{\lambda}_1-\lambda_1\right)\mathrm{Im}\,\lambda_1}, & w_2^{(0,3)} &= -\frac{i\kappa}{8\bar{\lambda}_1\mathrm{Im}\,\lambda_1},\\
w_3^{(0,3)} &= \frac{i\kappa}{4\left(3\bar{\lambda}_1-\lambda_3\right)\mathrm{Im}\,\lambda_3}, & w_4^{(0,3)} &= -\frac{i\kappa}{4\left(3\bar{\lambda}_1-\bar{\lambda}_3\right)\mathrm{Im}\,\lambda_3},
\end{aligned}$$

$$w_1^{(2,1)}=0, \quad w_2^{(2,1)}=\frac{-i3\kappa}{8\lambda_1\mathrm{Im}\,\lambda_1}, \quad w_3^{(2,1)}=\frac{i3\kappa}{4\left(2\lambda_1+\bar{\lambda}_1-\lambda_3\right)\mathrm{Im}\,\lambda_3}, \quad w_4^{(2,1)}=\frac{-i3\kappa}{4\left(2\lambda_1+\bar{\lambda}_1-\bar{\lambda}_3\right)\mathrm{Im}\,\lambda_3},$$

$$w_1^{(1,2)}=\frac{i3\kappa}{8\bar{\lambda}_1\mathrm{Im}\,\lambda_1}, \quad w_2^{(1,2)}=0, \quad w_3^{(1,2)}=\frac{i3\kappa}{4\left(\lambda_1+2\bar{\lambda}_1-\lambda_3\right)\mathrm{Im}\,\lambda_3}, \quad w_4^{(1,2)}=\frac{-i3\kappa}{4\left(\lambda_1+2\bar{\lambda}_1-\bar{\lambda}_3\right)\mathrm{Im}\,\lambda_3},$$

$$\beta_1=\frac{i3\kappa}{4\mathrm{Im}\,\lambda_1}.$$

The coefficients for $\ell=2$ are the complex conjugates of the above. The transformation for the SSM $W(\mathcal{E}_1)$ up to cubic order is therefore of the form

$$\boldsymbol{W}(z_1,\bar{z}_1)=\begin{pmatrix}
z_1+\frac{i\kappa z_1^3}{8\lambda_1\mathrm{Im}\,\lambda_1}+\frac{i\kappa\bar{z}_1^3}{4\left(3\bar{\lambda}_1-\lambda_1\right)\mathrm{Im}\,\lambda_1}+\frac{i3\kappa z_1\bar{z}_1^2}{8\bar{\lambda}_1\mathrm{Im}\,\lambda_1}\\
\bar{z}_1-\frac{i\kappa z_1^3}{4\left(3\lambda_1-\bar{\lambda}_1\right)\mathrm{Im}\,\lambda_1}-\frac{i\kappa\bar{z}_1^3}{8\bar{\lambda}_1\mathrm{Im}\,\lambda_1}-\frac{i3\kappa z_1^2\bar{z}_1}{8\lambda_1\mathrm{Im}\,\lambda_1}\\
\frac{i\kappa z_1^3}{4(3\lambda_1-\lambda_3)\mathrm{Im}\,\lambda_3}+\frac{i\kappa\bar{z}_1^3}{4\left(3\bar{\lambda}_1-\lambda_3\right)\mathrm{Im}\,\lambda_3}+\frac{i3\kappa z_1^2\bar{z}_1}{4\left(2\lambda_1+\bar{\lambda}_1-\lambda_3\right)\mathrm{Im}\,\lambda_1}+\frac{i3\kappa z_1\bar{z}_1^2}{4\left(\lambda_1+2\bar{\lambda}_1-\lambda_3\right)\mathrm{Im}\,\lambda_3}\\
-\frac{i\kappa z_1^3}{4\left(3\lambda_1-\bar{\lambda}_3\right)\mathrm{Im}\,\lambda_3}-\frac{i\kappa\bar{z}_1^3}{4\left(3\bar{\lambda}_1-\bar{\lambda}_3\right)\mathrm{Im}\,\lambda_3}-\frac{i3\kappa z_1^2\bar{z}_1}{4\left(2\lambda_1+\bar{\lambda}_1-\bar{\lambda}_3\right)\mathrm{Im}\,\lambda_3}-\frac{i3\kappa z_1\bar{z}_1^2}{4\left(\lambda_1+2\bar{\lambda}_1-\bar{\lambda}_3\right)\mathrm{Im}\,\lambda_3}
\end{pmatrix}. \tag{41}$$

Passing to polar coordinates via the substitution $z_1 = \rho_1 e^{i\theta_1}$, the corresponding leading-order dynamics (32)-(33) on $W(\mathcal{E}_1)$ is given by the equations

$$\begin{aligned} \dot{\rho}_1 &= -\frac{c}{2}\rho_1, && (42)\\ \dot{\theta}_1 &= \frac{1}{2}\sqrt{4k_0 - c^2} + \frac{3\kappa}{2\sqrt{4k_0 - c^2}}\rho_1^2. && (43) \end{aligned}$$

As obtained in formula (34), the instantaneous frequency of the oscillations on the SSM $\mathcal{E}_1$ is then

$$\omega(\rho_1) = \frac{1}{2}\left(\sqrt{4k_0 - c^2} + \frac{3\kappa}{\sqrt{4k_0 - c^2}}\rho_1^2\right).$$

The squared L^2 norm of the amplitude in the original x coordinates

$$\begin{aligned} [\mathrm{Amp}(\rho_1)]^2 &= \frac{1}{2\pi}\int_0^{2\pi} \left|\boldsymbol{V_x}\boldsymbol{W}(\rho_1 e^{i\theta_1}, \rho_1 e^{-i\theta_1})\right|^2 d\theta = \\ &= \frac{1}{2\pi}\int_0^{2\pi} \left|W_1 + \bar{W}_1 + W_3 + \bar{W}_3\right|^2 d\theta + \frac{1}{2\pi}\int_0^{2\pi} \left|W_1 + \bar{W}_1 - W_3 - \bar{W}_3\right|^2 d\theta \\ &= \frac{1}{2\pi}\int_0^{2\pi} \left|z_1 + \bar{z}_1 + \mathcal{O}\left(|\boldsymbol{z}|^3\right)\right|^2 d\theta + \frac{1}{2\pi}\int_0^{2\pi} \left|z_1 + \bar{z}_1 + \mathcal{O}\left(|\boldsymbol{z}|^3\right)\right|^2 d\theta \\ &= \frac{1}{2\pi}\int_0^{2\pi} \left[4z_1\bar{z}_1 + \mathcal{O}\left(|\boldsymbol{z}|^4\right)\right] d\theta = 4\rho_1^2 + \mathcal{O}\left(\rho_1^4\right), \end{aligned}$$

which gives $\mathrm{Amp}(\rho_1) \approx 2\rho_1$ for small ρ_1. For higher values of ρ_1, the exact dependence form of $\mathrm{Amp}(\rho_1)$ can be found by evaluating the above integral numerically.

Similarly, for $\ell = 3$, Theorem 1 (main text) gives the coefficients

$$w_3^{(1,0)} = 1, \quad w_1^{(1,0)} = w_2^{(1,0)} = w_4^{(1,0)} = 0, \quad w_4^{(0,1)} = 1, \quad w_1^{(0,1)} = w_2^{(0,1)} = w_3^{(0,1)} = 0,$$
$$w_j^{(2,0)} = w_j^{(1,1)} = w_j^{(0,2)} = 0, \qquad j = 1,2,3,4,$$

$$\begin{aligned} w_1^{(3,0)} &= \frac{i\kappa}{4\left(3\lambda_3 - \lambda_1\right)\mathrm{Im}\,\lambda_1}, \quad w_2^{(3,0)} = -\frac{i\kappa}{4\left(3\lambda_3 - \bar{\lambda}_1\right)\mathrm{Im}\,\lambda_1}, \\ & w_3^{(3,0)} = \frac{i\kappa}{8\lambda_3\mathrm{Im}\,\lambda_3}, \quad w_4^{(3,0)} = -\frac{i\kappa}{4\left(3\lambda_3 - \bar{\lambda}_3\right)\mathrm{Im}\,\lambda_3}, \\ w_1^{(0,3)} &= \frac{i\kappa}{4\left(3\bar{\lambda}_3 - \lambda_1\right)\mathrm{Im}\,\lambda_1}, \quad w_2^{(0,3)} = -\frac{i\kappa}{4\left(3\bar{\lambda}_3 - \bar{\lambda}_1\right)\mathrm{Im}\,\lambda_1}, \\ & w_3^{(0,3)} = \frac{i\kappa}{4\left(3\bar{\lambda}_3 - \lambda_3\right)\mathrm{Im}\,\lambda_3}, \quad w_4^{(0,3)} = -\frac{i\kappa}{8\bar{\lambda}_3\mathrm{Im}\,\lambda_3}, \end{aligned}$$

$$w_1^{(2,1)} = \frac{i3\kappa}{4\left(2\lambda_3 + \bar{\lambda}_3 - \lambda_1\right)\mathrm{Im}\,\lambda_1}, \quad w_2^{(2,1)} = \frac{-i3\kappa}{4\left(2\lambda_3 + \bar{\lambda}_3 - \bar{\lambda}_1\right)\mathrm{Im}\,\lambda_1}, \quad w_3^{(2,1)} = 0, \quad w_4^{(2,1)} = \frac{-i3\kappa}{8\lambda_3\mathrm{Im}\,\lambda_3},$$

$$w_1^{(1,2)} = \frac{i3\kappa}{4\left(\lambda_3 + 2\bar{\lambda}_3 - \lambda_1\right)\mathrm{Im}\,\lambda_1}, \quad w_2^{(1,2)} = \frac{-i3\kappa}{4\left(\lambda_3 + 2\bar{\lambda}_3 - \bar{\lambda}_1\right)\mathrm{Im}\,\lambda_1}, \quad w_3^{(1,2)} = \frac{i3\kappa}{8\bar{\lambda}_3\mathrm{Im}\,\lambda_3}, \quad w_4^{(1,2)} = 0,$$

$$\beta_3 = \frac{i3\kappa}{4\mathrm{Im}\,\lambda_3},$$

whose complex conjugates are the corresponding coefficients for $\ell = 4$.

Similarly, the transformation for the SSM $W(\mathcal{E}_2)$ up to cubic order is of the form

$$\boldsymbol{W}(z_3, \bar{z}_3) = \begin{pmatrix} \frac{i\kappa z_3^3}{4(3\lambda_3-\lambda_1)\mathrm{Im}\,\lambda_1} + \frac{i\kappa \bar{z}_3^3}{4\left(3\bar{\lambda}_3-\lambda_1\right)\mathrm{Im}\,\lambda_1} + \frac{i3\kappa z_3^2\bar{z}_3}{4\left(2\lambda_3+\bar{\lambda}_3-\lambda_1\right)\mathrm{Im}\,\lambda_1} + \frac{i3\kappa z_3\bar{z}_3^2}{4\left(\lambda_3+2\bar{\lambda}_3-\lambda_1\right)\mathrm{Im}\,\lambda_1} \\ -\frac{i\kappa z_3^3}{4\left(3\lambda_3-\bar{\lambda}_1\right)\mathrm{Im}\,\lambda_1} - \frac{i\kappa \bar{z}_3^3}{4\left(3\bar{\lambda}_3-\bar{\lambda}_1\right)\mathrm{Im}\,\lambda_1} - \frac{i3\kappa z_3^2\bar{z}_3}{4\left(2\lambda_3+\bar{\lambda}_3-\bar{\lambda}_1\right)\mathrm{Im}\,\lambda_1} - \frac{i3\kappa z_3\bar{z}_3^2}{4\left(\lambda_3+2\bar{\lambda}_3-\bar{\lambda}_1\right)\mathrm{Im}\,\lambda_1} \\ z_3 + \frac{i\kappa z_3^3}{8\lambda_3\mathrm{Im}\,\lambda_3} + \frac{i\kappa \bar{z}_3^3}{4\left(3\bar{\lambda}_3-\lambda_3\right)\mathrm{Im}\,\lambda_3} + \frac{i3\kappa z_3\bar{z}_3^2}{8\bar{\lambda}_3\mathrm{Im}\,\lambda_3} \\ \bar{z}_3 - \frac{i\kappa z_3^3}{4\left(3\lambda_3-\bar{\lambda}_3\right)\mathrm{Im}\,\lambda_3} - \frac{i\kappa \bar{z}_3^3}{8\bar{\lambda}_3\mathrm{Im}\,\lambda_3} - \frac{i3\kappa z_3^2\bar{z}_3}{8\lambda_3\mathrm{Im}\,\lambda_3} \end{pmatrix}, \tag{44}$$

with the corresponding dynamics on the first SSM is described by (cf. (32)-(33))

$$\dot{\rho}_3 = -\frac{3c}{2}\rho_3, \tag{45}$$

$$\dot{\theta}_3 = \frac{1}{2}\sqrt{3\left(4k_0 - 3c^2\right)} + \frac{\sqrt{3}\kappa}{2\sqrt{4k_0-3c^2}}\rho_3^2. \tag{46}$$

Following the same calculation as for $\ell = 1$, we obtain

$$\omega(\rho_3) = \frac{1}{2}\left(\sqrt{3\left(4k_0-3c^2\right)} + \frac{\sqrt{3}\kappa}{\sqrt{4k_0-3c^2}}\rho_3^2\right),$$

$$\mathrm{Amp}(\rho_3) \approx 2\rho_3.$$

References

[1] X. Cabre, E. Fontich, and R. de la Llave. The parameterization method for invariant manifolds I: Manifolds associated to non-resonant subspaces. *Indiana Univ. Math. J.*, 52:283–328, 2003.

[2] G. Haller and S. Ponsioen. Nonlinear normal modes and spectral submanifolds: existence, uniqueness and use in model reduction. *Nonlinear Dynamics*, pages 1–42, 2016. `doi:10.1007/s11071-016-2974-z`.
